# Metabolic disorder and intestinal microflora dysbiosis in chronic inflammatory demyelinating polyradiculoneuropathy

**DOI:** 10.1186/s13578-023-00956-1

**Published:** 2023-01-11

**Authors:** Jiafang Fu, Jingli Shan, Yazhou Cui, Chuanzhu Yan, Qinzhou Wang, Jinxiang Han, Guangxiang Cao

**Affiliations:** 1 Biomedical Sciences College & Shandong Medicinal Biotechnology Centre, First Affiliated Hospital of Shandong First Medical University, Shandong First Medical University & Shandong Academy of Medical Sciences, Jinan, 250117 China; 2 Research Institute of Neuromuscular and Neurodegenerative Diseases and Department of Neurology, Qilu Hospital, Cheeloo College of Medicine, Shandong University, Jinan, 250012 China; 3 Key Lab for Rare & Uncommon Diseases of Shandong Province, Jinan, 250117 China; 4 NHC Key Laboratory of Biotechnology Drugs, Shandong Academy of Medical Sciences, Jinan, 250117 China; 5 Department of Central Laboratory and Mitochondrial Medicine Laboratory, Qilu Hospital (Qingdao), Cheeloo College of Medicine, Shandong University, Qingdao, 266035 China; 6 Brain Science Research Institute, Shandong University, Jinan, 250012 China

**Keywords:** Chronic inflammatory demyelinating polyradiculoneuropathy, Bile acids, Arachidonic acid, Metabolic disorder, Gut microbial dysbiosis

## Abstract

**Objective:**

Chronic inflammatory demyelinating polyradiculoneuropathy (CIDP) is a rare acquired immune-mediated neuropathy. Although microbial infection is potentially a contributing factor, a causative link between CIDP and microbial infection remains unclear. There is also no definitive biomarker for CIDP diagnostics and therapies. The present study aimed to characterize the serum metabolic profile and gut microbiome structure in CIDP.

**Methods:**

Targeted metabolomics profiling of serum, using liquid chromatography-mass spectrometry, and metagenomics sequencing of stool samples from a cohort of CIDP and non-CIDP subjects were performed to evaluate serum metabolic profiles and gut microbiome structure in CIDP subjects relative to healthy controls.

**Results:**

Metabolome data revealed that the bile acids profile was perturbed in CIDP with bile acids and arachidonic acid enriched significantly in CIDP versus non-CIDP controls. Metagenome data revealed that opportunistic pathogens, such as *Klebsiella pneumonia* and *Megamonas funiformis*, and genes involved in bacterial infection were notably more abundant in CIDP subjects, while gut microbes related to biotransformation of secondary bile acids were abnormal in CIDP versus non-CIDP subjects. Correlation analysis revealed that changes in secondary bile acids were associated with altered gut microbes, including *Bacteroides ovatus*, *Bacteroides caccae*, and *Ruminococcus gnavus*.

**Conclusion:**

Bile acids and arachidonic acid metabolism were disturbed in CIDP subjects and might be affected by the dysbiosis of gut microbial flora. These findings suggest that the combination of bile acids and arachidonic acid could be used as a CIDP biomarker and that modulation of gut microbiota might impact the clinical course of CIDP.

**Supplementary Information:**

The online version contains supplementary material available at 10.1186/s13578-023-00956-1.

## Introduction

Chronic inflammatory demyelinating polyradiculoneuropathy (CIDP) is a rare, acquired, and immune-mediated demyelinating neuropathy causing limb weakness and sensory deficits [1]. The cause of CIDP remains unknown, and diagnosis is challenging. The disease course is steadily progressive for more than 8 weeks and is characterized by strong heterogeneity in terms of clinical presentation, prognosis, and treatment response [2]. CIDP diagnosis is usually based on a progressive or relapsing course over 2 months, electrophysiological evidence of peripheral demyelination, as well as response to immune-modulating therapies [3–5]. Auto-antibodies against ganglioside proteins [6] and autoreactive T-cell responses against myelin antigens [7, 8] are reported to be involved in the immunopathogenesis of CIDP, indicating different autoimmune targets are likely to be relevant. However, none of the immunopathological findings are specific for CIDP, meaning that the results of the diagnostic tests should be carefully interpreted as misdiagnosis commonly occurs. In addition, monitoring disease activity in order to guide treatment management is also a difficult clinical problem, as nerve conduction studies and electromyography do not adequately reflect functional disability, especially in severe disease courses [9, 10]. Hence, there is an urgent need for providing valid biomarkers to diagnose CIDP.

Another type of immune-mediated demyelinating neuropathy, Guillain Barre syndrome (GBS), also named acute inflammatory demyelinating polyradiculoneuropathy, was reported to be related to microbial infection [11, 12]. The post-infectious nature of GBS is today well-recognized in the majority of presenting cases, and approximately 70% of GBS cases are preceded by an infectious respiratory or gastrointestinal illness [13]. Molecular mimicry between microbial and axolemmal surface molecules after *Campylobacter jejuni* infection is known to be the pathophysiological basis of neural involvement leading to GBS [14, 15]. In contrast to GBS, data on preceding infections for CIDP are not only lacking but also heterogeneous. Earlier studies indicated that up to 30% of CIDP cases are associated with preceding infection [16–19], while more recent studies suggested 10% to 20% [20, 21]. However, the causative link between CIDP and microbial infection remains unproven.

To address the above knowledge gap, an unbiased approach was taken to identify serum metabolites and gut microbial flora that were significantly changed in CIDP subjects relative to non-CIDP controls. To that end, untargeted metabolomics profiling of serum, using liquid chromatography-mass spectrometry (LC–MS), was performed to discover potential biomarkers for CIDP, and metagenomics analysis of gut microbial species and genes in stool samples was performed to discover the association between CIDP and gut microbial flora. Additionally, Pearson’s correlation analysis was performed to explore the association between the bile acids (BAs) profile and the microbiotic abundance in the gut. Our findings provide an improved understanding of perturbations of the metabolome-microbiome interface in CIDP, including identification of potential biomarkers to monitor ongoing CIDP activity and modulation of gut microbiota to prevent CIDP.

## Methods

### Study population and sample handling

A total of 64 subjects, including 31 CIDP patients and 33 age- and sex-matched healthy controls (Additional file 1: Table S1), were enrolled in this study from December 2020 to February 2022 at the Qilu Hospital of Shandong University. Diagnosis of CIDP was based on established guidelines for diagnosis and treatment of CIDP [3, 22]. Serum and stool samples were collected from subjects, immediately immersed in liquid nitrogen, and then frozen at − 80 °C. All samples were kept frozen until further analysis.

### Metabolite extraction

Metabolites were extracted from serum essentially according to previously reported methods [23, 24]. In short, samples were extracted by adding precooled methanol and acetonitrile (2:1, v/v), and internal standards mixes 1 and 2 were added for quality control of sample preparation. After purified, the metabolites were prepared for LC-MS analysis.

### LC–MS/MS analysis

The untargeted LC-MS/MS used a Waters 2D UPLC (Waters, USA) tandem Q Exactive high-resolution mass spectrometer (Thermo Fisher Scientific, USA) for separation and detection of metabolites. Chromatographic separation was performed on a Waters ACQUITY UPLC BEH C18 column (1.7 μm, 2.1 mm × 100 mm), with a column temperature of 45 °C. The mobile phase consisted of 0.1% formic acid and acetonitrile in the positive mode and 10 mM ammonium formate and acetonitrile in the negative mode. The mass spectrometric settings for positive or negative ionization modes were as follows: spray voltage, 3.8/ − 3.2 kV; sheath gas flow rate, 40 arbitrary units (arb); aux gas flow rate, 10 arb; aux gas heater temperature, 350 °C; capillary temperature, 320 °C. The full scan range was 70–1050 m/z with a resolution of 70,000, and the automatic gain control (AGC) target for MS acquisitions was set to 3e6 with a maximum ion injection time of 100 ms.

### Principal component analysis (PCA) analysis of metabolites

A PCA model [25] was established for the comparative analysis of CIDP group and non-CIDP group to observe the distribution and separation trends between samples. The data were subjected to log2 conversion before the PCA model was established, with scaling by the Pareto scaling method.

### Partial least squares-discriminant analysis (PLS-DA) analysis of metabolites

A PLS-DA [26] model between the comparative CIDP group and non-CIDP group was established after log2-log conversion of the metabolite data, using the Par scaling method and sevenfold cross validation. The PLS-DA model [26] was subjected to 200 response permutation tests. Variable importance in projection (VIP) was used to measure the influence intensity and interpretation ability of each metabolite, and variables with a VIP greater than 1 were considered to have a significant effect on the classification of sample categories.

### DNA extraction and metagenomics sequencing and de novo assembly

Metagenomic extraction and metagenomics sequencing were performed at the BGI Co., Ltd. (Shenzhen, China). Stool metagenomic DNA extractions were performed using the MolPure® Stool DNA Kit (YESEN, Shanghai, China). Metagenomic libraries were sequenced on the DNBSEQ platform. High-quality short reads of each DNA sample were assembled by MEGAHIT [27].

### Gene prediction and functional annotation

MetaGeneMark [28] was used for ab initio prediction of metagenomic genes. Functional annotations were made using BacMet [29] (20180311), KEGG [30] (v101) databases, COG [31] (20201125), and SwissProt [32] (release-2021_04).

### Non-metric multidimensional scale (NMDS) analysis and analysis of similarities (ANOSIM)

NMDS was conducted as previously described [33], and ANOSIM was conducted by the “vegan” packages of R (v3.3.1) based on Bray-Curtis distance [34]. Species diversity analysis was conducted as previously described [35].

### Statistical analyses

All statistical analyses were performed using GraphPad Prism 9.0 software. A two-tailed *t*-test was performed to compare the CIDP and non-CIDP groups. Correlations between the gut microbial abundance and BAs profile were estimated using Pearson’s correlation analysis.

## Results

### Broad metabolic shifts in CIDP Patients

**Fig. 1:**
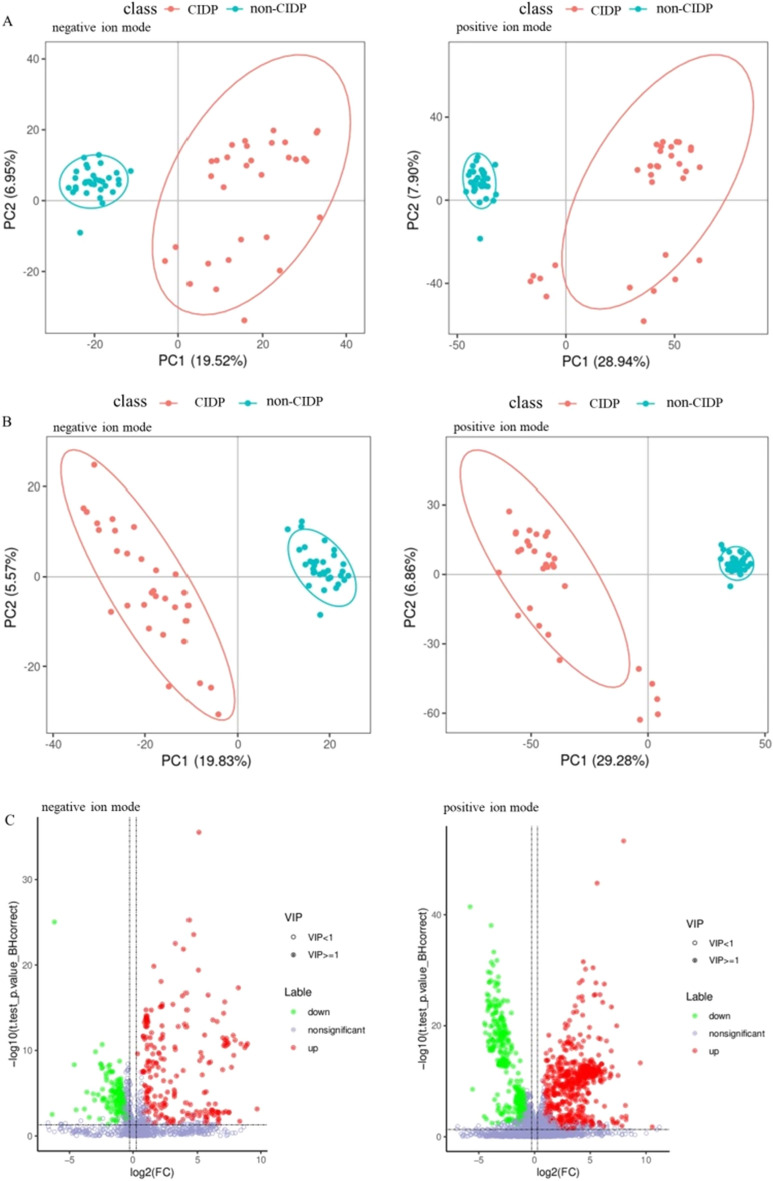
Multivariate statistical analysis and screening of differential metabolites. **A** PCA score map for negative or positive ion mode data. Each dot represents a sample, and different groups are labeled with different colors. **B** PLS-DA score map for negative or positive ion mode data. The number in parentheses is the score for the principal component, which represents the percentage of the explanation on overall variance of the specific principal component. **C** Volcano map of differential metabolites in negative or positive ion mode. Green dots and red dots represent down-regulated and up-regulated differential metabolites in CIDP group, respectively, and metabolites without differences are labeled purple-gray

To characterize the metabolomic profile in CIDP, each serum sample was analyzed by LC–MS/MS in non-targeted mode, and MS/MS was performed using sensitive, high-resolution MS to collect data from both positive and negative ionization modes to improve metabolite coverage. A total of 4739 different metabolites were detected in positive ion mode, among which 1736 metabolites were identified, and 1504 different metabolites were detected in negative ion mode, with 744 metabolites identified. To determine whether there were significant differences in metabolites between the CIDP and non-CIDP groups, a PCA model was established to observe the distribution and separation trend of the two groups of samples (Fig. 1A), and PLS-DA was also performed (Fig. 1B). Both the PCA and PLS-DA models showed that the major patterns of serum metabolites were largely separated for CIDP patients versus non-CIDP controls.

Further statistical analysis revealed that 597 metabolites identified in positive ion mode increased in CIDP compared with non-CIDP, and 481 metabolites decreased; additionally, 208 metabolites increased in CIDP, and 182 metabolites decreased in negative ion mode, indicating broad metabolic shifts in CIDP versus non-CIDP controls, as also demonstrated by the volcano plots (Fig. 1C).

### Enrichments in bile acids and arachidonic acid in CIDP

Quantification and comparison of serum metabolites identified some with significant changes in CIDP patients (Additional file 1: Table S2 and S3). Analysis of the enriched serum metabolites revealed that BAs changed significantly in the CIDP group versus non-CIDP controls (Table 1). For primary BAs, the concentration of cholic acid (CA) was markedly increased in the CIDP group by 9.61-fold and 36.46-fold, respectively, in the negative and positive ion modes. Concentrations of taurocholic acid (TCA) and taurochenodeoxycholic acid 3-sulfate (TCDCS) increased by 38.30-fold and about eightfold, and only glycocholic acid (GCA) decreased in the CIDP group. Among secondary BAs, deoxycholic acid (DCA) and taurodeoxycholic acid (TDCA) were increased in the CIDP group, while tauroursodeoxycholic acid (TUDCA), glycodeoxycholic acid (GDCA), lithocholic acid taurine conjugate (TLCA), and taurolithocholic acid 3-sulfate (TLCS) were lower in the CIDP group than in the non-CIDP group, indicating that secondary BA metabolic pathways that associated with by microorganisms were abnormal in CIDP versus non-CIDP subjects.

**Table 1:** BA and Arachidonic Acid Profiles in CIDP versus Non-CIDP Subjects

Class	Metabolite	Fold-change	T-test_p-value	VIP	Ion mode
Host-derived primary BAs	CA	9.61/36.46	0/0.0001	1.61/1.51	N/P^a^
	TCA	38.30	0	2.46	P
	GCA	0.55/0.57	0.0005/0.0006	1.13/0.95	N/P
	TCDCS	8.87/7.81	0/0	2.46/2.06	N/P
	GCDCA	0.46/0.42	0/0	1.23/1.12	N/P
Microbial- derived secondary BAs	DCA	1.93/4.69	0.055/0.2405	0.72/0.62	N/P
	TDCA	14.66	0	2.09	P
	GDCA	0.43	0.0466	0.74	N
	TUDCA	0.70	0.0035	0.97	N
	TLCA	0.24	0	1.58	N
	TLCS	0.65	0.1243	0.59	N
Derivative of BA	DHCA	47.63	0	2.90	N
Arachidonic acid and its related metabolites	AA	2.00	0	1.15	N
	TXB2	41.71	0	2.42	N
	linoleic acid	1.76	0.0047	0.80	N
	8z,11z,14z-eicosatrienoic acid	1.75/1.34	0.0001/0.0167	0.91/0.005	N/P

^a^N represents negative ion mode, and P represents positive ion mode

**Fig. 2:**
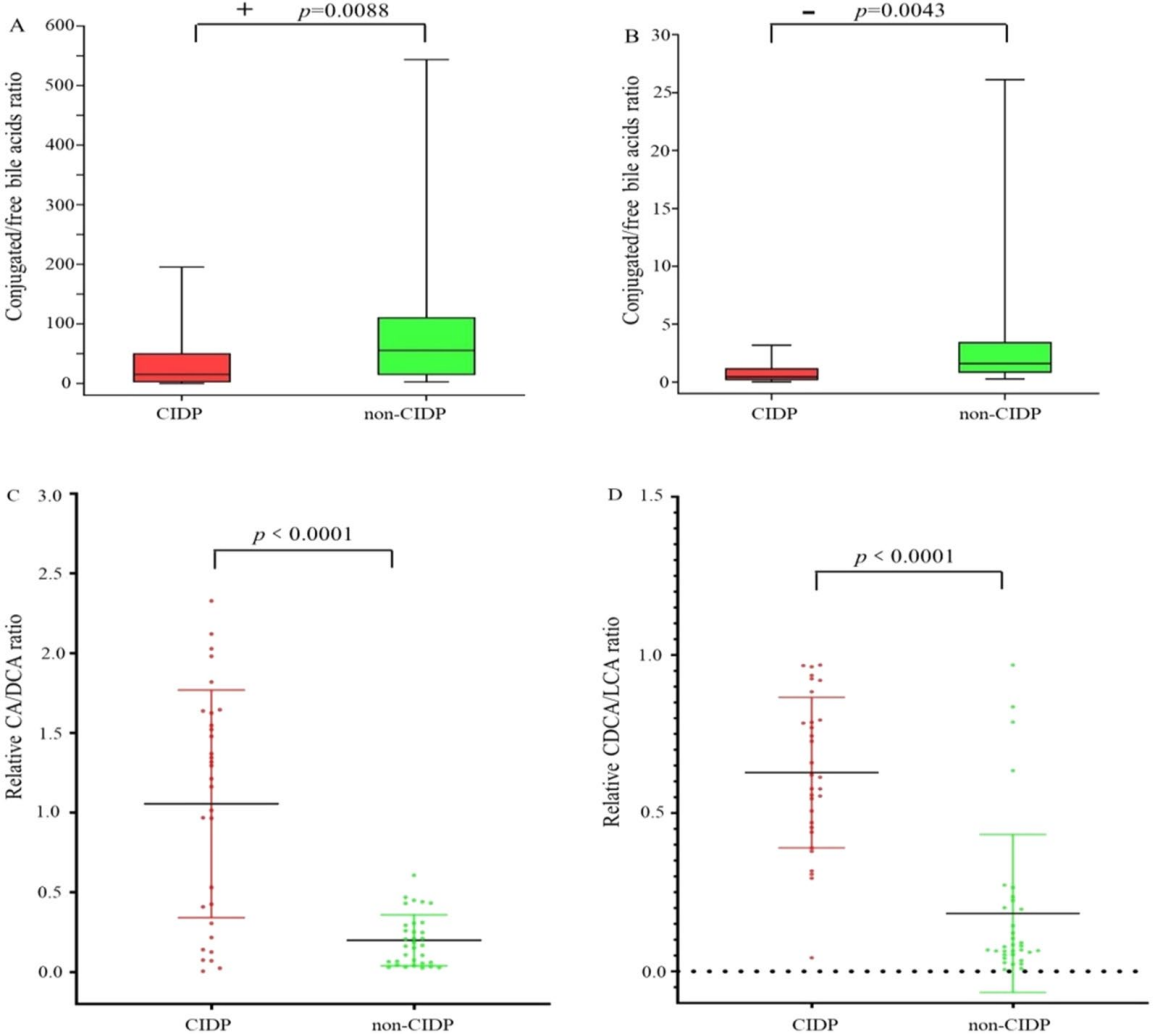
Comparisons of BA composition in the serum of CIDP versus non-CIDP subjects. **A**, **B** Boxplots for ratio of conjugated BAs to free BAs in **A** positive ion mode and **B** negative ion mode. **C** Comparisons of the relative CA-to-DCA ratios in CIDP versus non-CIDP serum. Relative CA value includes conjugated and free CA; relative DCA value includes conjugated and free DCA. **D** Comparisons of the relative CDCA-to-LCA ratio in CIDP versus non-CIDP serum. Relative CDCA value includes conjugated and free CDCA; relative LCA value includes conjugated and free LCA. Data are shown as mean ± SEM

We also analyzed the ratio of conjugated BAs to free BAs in the serum (Fig. 2A, B), and results showed that this ratio was lower in the CIDP group than in the non-CIDP group, indicating that host BA homeostasis was disrupted in CIDP. Since DCA is the microbial metabolic product of CA, and lithocholic acid (LCA) is the microbial metabolic product of chenodeoxycholic acid (CDCA), we calculated the relative CA-to-DCA ratio and relative CDCA-to-LCA ratio in the CIDP and non-CIDP groups to indirectly evaluate the effects of the gut microbiota. Results showed notable increases in the CA/DCA and CDCA/LCA ratios in the serum of the CIDP group (Fig. 2C, D). Our results indicate that CIDP markedly inhibited intestinal secondary BA formation.

**Fig. 3:**
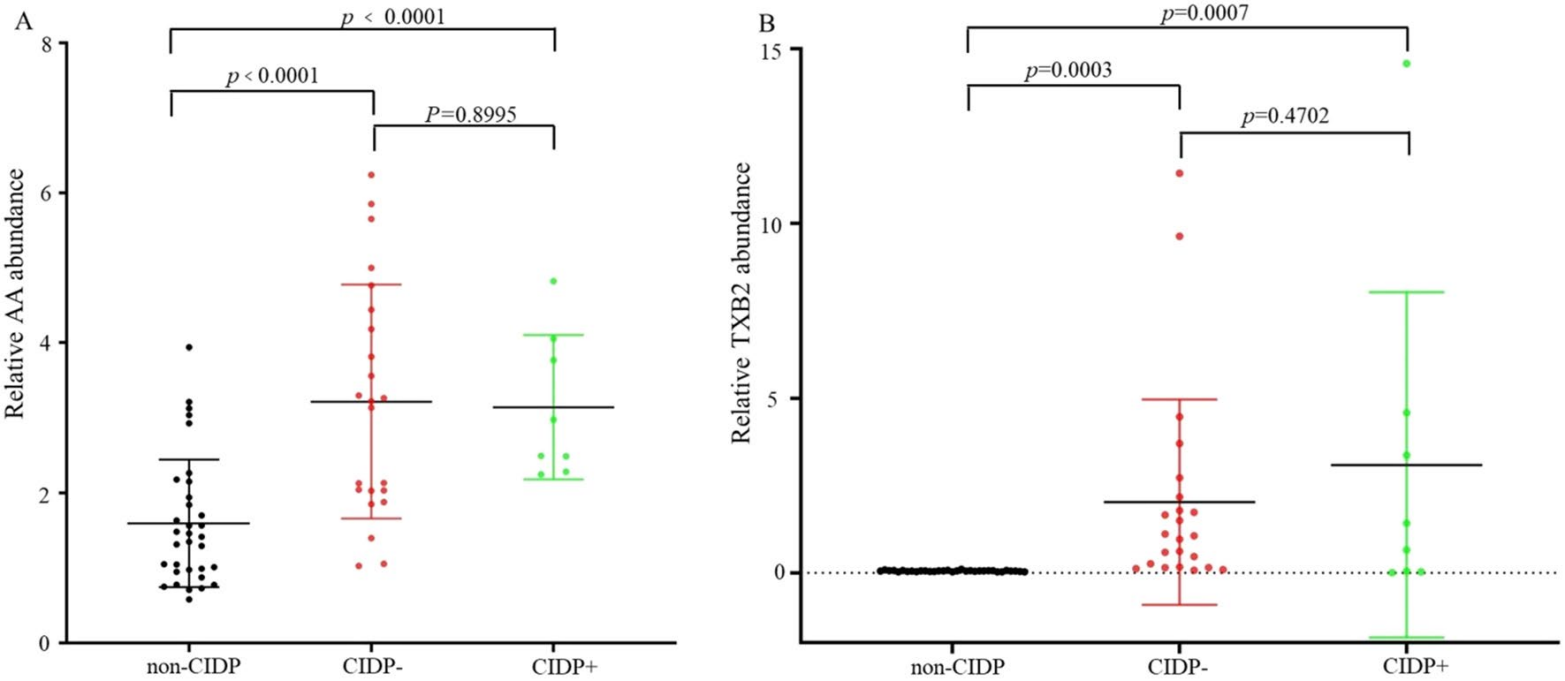
Comparisons of the relative abundance of AA and its mediator TXB2. **A** Relative AA abundance and **B** relative TXB2 abundance in the serum of non-CIDP, CIDP- (without cardiovascular disease), and CIDP + (with cardiovascular disease) groups

Arachidonic acid (AA) and the mediator in AA metabolism (thromboxane b2, TXB2) are reported to be involved in different neurological disorders and cardiovascular diseases [36–38]. In this study, metabolomics data showed that AA increased by about twofold and TXB2 increased by about 42-fold in CIDP versus non-CIDP subjects and that the precursors of AA (linoleic acid and 8z, 11z, 14z-eicosatrienoic acid) also increased (Table 1). As some CIDP subjects developed cardiovascular disease (Additional file 1: Table S1), we further evaluated whether the increased AA and TXB2 were associated with cardiovascular disease. However, statistical analysis revealed no significant differences in the relative abundance of AA (*p* = 0.8995) and TXB2 (*p* = 0.4702) in CIDP patients with or without cardiovascular disease, whereas the relative abundance of AA (*p* < 0.0001 and *p* < 0.0001) and TXB2 (*p* = 0.0007 and *p* = 0.0003) differed significantly between the non-CIDP group and CIDP group regardless of the presence of cardiovascular disease (Fig. 3), indicating an association between CIDP and significantly elevated levels of AA and TXB2.

### Metabolic pathways are perturbed in CIDP subjects

**Fig. 4:**
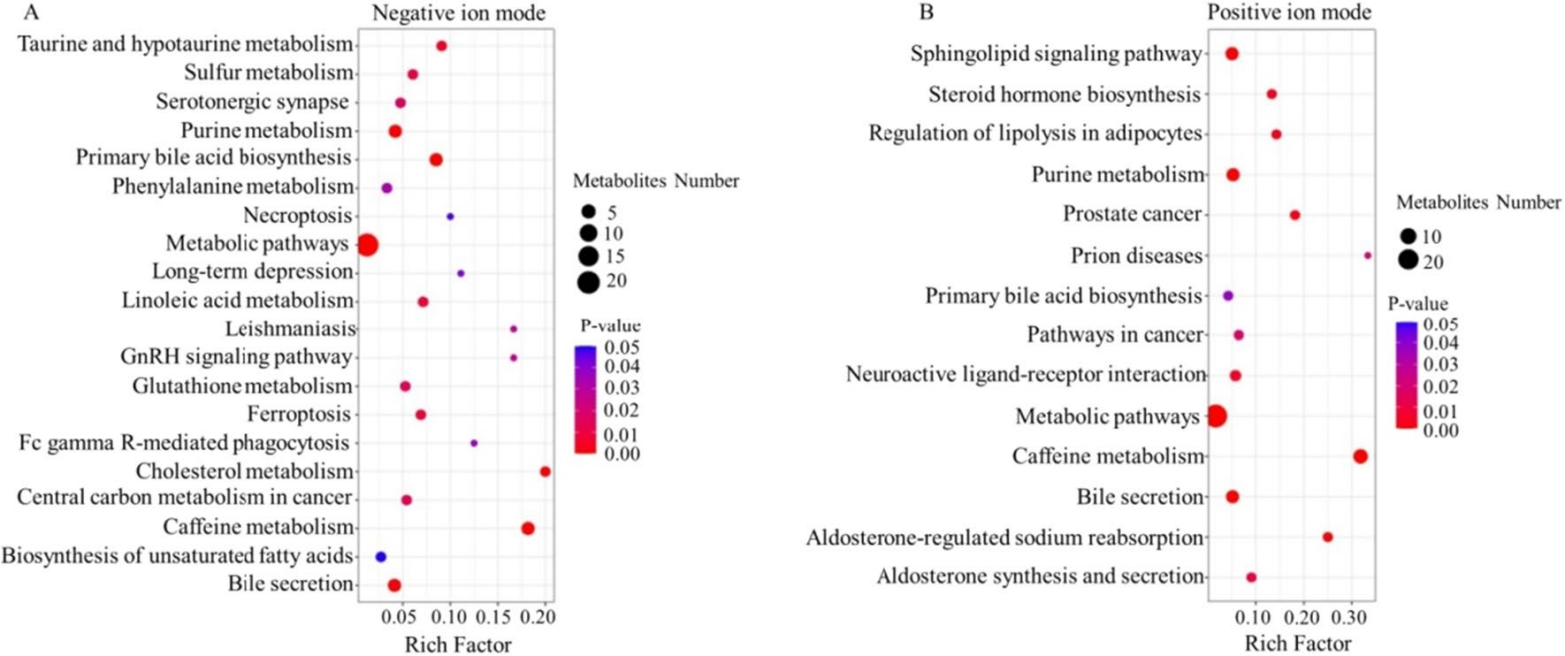
Bubble plots for metabolic pathway enrichment analysis. Differentially abundant metabolites identified in the negative ion mode **A** and in the positive ion mode **B**. X-axis enrichment factor (Rich Factor) denotes the number of differentially metabolites annotated to the pathway divided by all identified metabolites annotated to the pathway. The larger the value, the greater the proportion of differentially metabolites annotated to the pathway. The dot size indicates the number of differentially metabolites annotated to a given pathway

Metabolic pathway enrichment analysis was performed based on the KEGG database, and differentially abundant metabolites and their pathways identified in negative and positive ion modes were evaluated (Additional file 1: Table S2 and Table S3). Results showed that 20 metabolic pathways presented significant differences in CIDP compared with non-CIDP in the negative ion mode, and 14 metabolic pathways were perturbed in the positive ion mode (Fig. 4). The metabolites that differed in abundance between the CIDP and non-CIDP groups were mainly involved in primary BA biosynthesis, bile secretion, linoleic acid metabolism, and taurine and hypotaurine metabolism, with BA and AA appearing most frequently in the differentially enriched metabolic pathways (Fig. 4), indicating that BA and AA might be two key metabolites for CIDP.

### Gut microbiome composition changes in CIDP

In this study, 28 qualified stool samples from 31 CIDP subjects and 33 qualified stool samples from non-CIDP subjects were obtained and tested. To determine whether the sample size of stool samples was reasonable, a rarefaction curve was drawn with sample (randomly sampled) size and taxonomic abundance of the samples. The rarefaction curve of CIDP and non-CIDP group tended to be steady (Additional file 1: Fig S1), indicating that the sampling quantities for the CIDP and non-CIDP groups were reasonable and sufficient. In addition, both ANOSIM and NMDS statistical analyses showed that the inter-group difference between CIDP and non-CIDP group was greater than the intra-group difference at the family level, genus level, and species level (Additional file 1: Fig S2), indicating that grouping was significant.

**Fig. 5:**
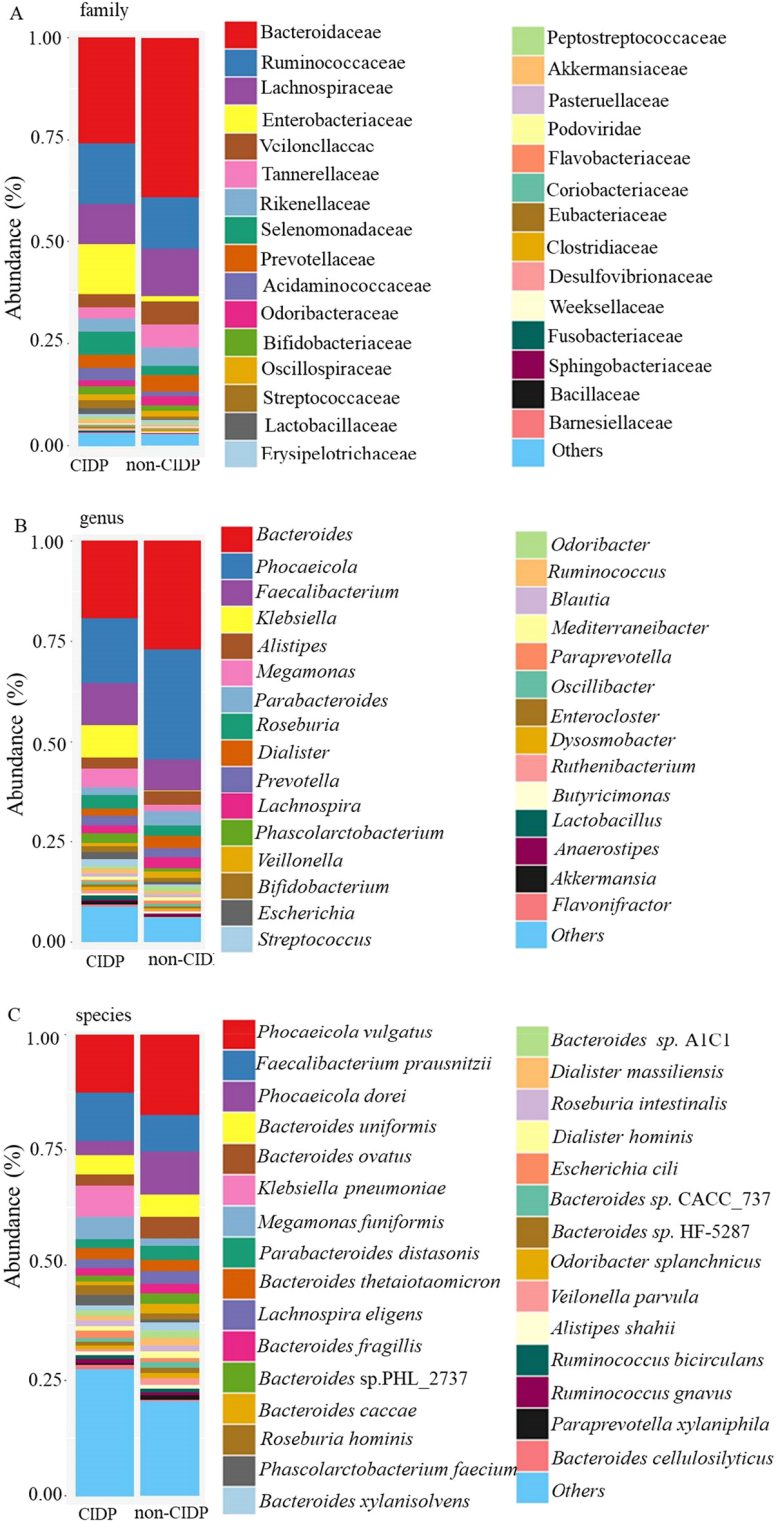
Taxonomic classifications and ratios of gut microbial flora in CIDP and non-CIDP groups. Microbiotic abundance is shown by taxonomy barplot at the family level **A**, genus level **B**, and species level **C**

Microbiotic abundance at the family level showed that Enterobacteriaceae, Selenomonadaceae and Acidaminococcaceae was significantly higher in group CIDP than in group non-CIDP, while Bacteroidaceae, Veillonellaceae, Tannerellaceae and Rikenellaceae were decreased in group CIDP (Fig. 5A). At the genus level, the most enriched genera in group CIDP were *Klebsiella* (Enterobacteriaceae), *Megamonas* (Selenomonadaceae), and *Phascolarctobacterium* (Acidaminococcaceae), while *Bacteroides* (Bacteroidaceae) and *Phocaeicola* (Bacteroidaceae) had reduced abundance (Fig. 5B). At the species level, the most enriched species in group CIDP were *Klebsiella pneumonia* (Enterobacteriaceae), *Escherichia coli* (Enterobacteriaceae), *Megamonas funiformis* (Selenomonadaceae), and *Phascolarctobacterium faecium* (Acidaminococcaceae), while *Phocaeicola dorei* (Bacteroidaceae), *Phocaeicola vulgatus* (Bacteroidaceae), *Bacteroides uniformis* (Bacteroidaceae), and *Bacteroides* sp. A1C1 (Bacteroidaceae) decreased (Fig. 5C). Overall, the microbiotic abundance data revealed gut microflora dysbiosis in the CIDP group, since potential pathogens such as *Klebsiella* were enriched, while indigenous microorganisms such as *Bacteroides* decreased.

Further analysis based on the microbe abundance in individual samples showed that almost all CIDP samples (except CIDP 32) had 1–3 types of above enriched microorganisms with levels higher than 1% at family, genus, and species levels, including *Klebsiella pneumoniae*, *Escherichia coli*, *Megamonas funiformis*, and *Phascolarctobacterium faecium* at levels > 1% in around 13 of the samples (Additional file 1: Fig S3). KEGG annotation of the metagenome also revealed that genes classified as associated with bacterial infection and disease were more abundant in CIDP subjects (Additional file 1: Fig S4). As *Klebsiella pneumonia*, *Escherichia coli,* and *Megamonas funiformis* are well-known opportunistic pathogens, these data indicate that CIDP may be related to intestinal microbial infection.

### Enriched genes involved in intestinal microbial infection in CIDP subjects

To further investigate whether there is the intestinal microbial pathogenic factor in CIDP, genes in metagenomes were annotated and analyzed. Consisting with the enrichment of opportunistic pathogens *Klebsiella pneumonia*, *Escherichia coli,* and *Megamonas funiformis* in CIDP, eighteen genes involved in bacterial infection, nine genes involved in bacterial invasion, three genes involved in quorum sensing were enriched in CIDP (Table 2), 70 genes involved in virulence factors were also enriched in CIDP (Additional file 1: Table S4). KEGG analysis revealed that pathways associated with infection such as bacterial invasion of epithelial cells, pathogenic bacteria infection, quorum sensing and bacterial secretion system, were enriched in CIDP groups (Additional file 1: Table S5). Previous study showed that type II secretion system [39], type III secretion system [40], type IV secretion system [41], type VI secretion system [42], and type VII secretion system [43, 44], played important roles during bacterial infection. In this study, other 259 genes involved in type II secretion system, type III secretion system, type IV secretion system and type VI secretion system were also enriched in CIDP (Additional file 1: Table S6). The enrichment of so many virulence factor-encoding genes and infection-related genes in CIDP further indicates that CIDP may be related to intestinal microbial infection.

**Table 2:** Genes involved in bacterial infection

Gene_id	Identity	E_value	Gene	Protein	Description
[Denovogenes]_330540	41	1.20E-11	Q09734	MIP_TRYCR	Macrophage infectivity potentiator
[Denovogenes]_425895	82.2	1.90E-88	P38396	SIEB_BPP22	Superinfection exclusion protein B
[Denovogenes]_443122	40.5	3.60E-12	Q09734	MIP_TRYCR	Macrophage infectivity potentiator
[Denovogenes]_660132	48.3	3.80E-25	Q09734	MIP_TRYCR	Macrophage infectivity potentiator
[Denovogenes]_697597	46.7	6.50E-14	Q09734	MIP_TRYCR	Macrophage infectivity potentiator
[Denovogenes]_768330	43.3	5.10E-21	Q09734	MIP_TRYCR	Macrophage infectivity potentiator
[Denovogenes]_769022	42.5	6.70E-21	Q09734	MIP_TRYCR	Macrophage infectivity potentiator
[Denovogenes]_906098	53.3	1.50E-16	Q09734	MIP_TRYCR	Macrophage infectivity potentiator
[Denovogenes]_917376	53.3	2.00E-16	Q09734	MIP_TRYCR	Macrophage infectivity potentiator
[Denovogenes]_920603	42.3	2.80E-15	P14109	VG17_BPP22	Superinfection exclusion protein
[Denovogenes]_922981	43.7	8.80E-17	P14109	VG17_BPP22	Superinfection exclusion protein
[Denovogenes]_1034820	81.4	7.30E-42	P14109	VG17_BPP22	Superinfection exclusion protein
[Denovogenes]_1329093	47.6	5.80E-15	Q09734	MIP_TRYCR	Macrophage infectivity potentiator
[Denovogenes]_1420535	46.4	8.10E-16	P14109	VG17_BPP22	Superinfection exclusion protein
[Denovogenes]_156709	41.5	4.70E-19	Q09734	MIP_TRYCR	Macrophage infectivity potentiator
[Denovogenes]_243359	43.8	6.10E-15	P22589	UBIQP_PHYIN	Polyubiquitin
[Denovogenes]_441162	79.8	1.10E-80	P26988	G3P_PHYIN	Glyceraldehyde-3-phosphate dehydrogenase
[Denovogenes]_703648	50.9	5.20E-19	P22589	UBIQP_PHYIN	Polyubiquitin
[Denovogenes]_253622	40.4	1.20E-34	P69342	INVF_SALTI	Invasion protein InvF
[Denovogenes]_591051	49.3	7.50E-35	E1WAC2	IAGB_SALTS	Invasion protein IagB
[Denovogenes]_606928	40.4	9.00E-17	E1WAC2	IAGB_SALTS	Invasion protein IagB
[Denovogenes]_607750	40.3	3.40E-16	E1WAC2	IAGB_SALTS	Invasion protein IagB
[Denovogenes]_856442	43.5	1.70E-15	P0CL15	IAGB_SALTY	Invasion protein IagB
[Denovogenes]_1028607	41.5	5.10E-11	E1WAC2	IAGB_SALTS	Invasion protein IagB
[Denovogenes]_1149013	43	3.50E-09	P43018	IAGB_SALTI	Invasion protein IagB
[Denovogenes]_1199611	48.4	1.60E-19	P43018	IAGB_SALTI	Invasion protein IagB
[Denovogenes]_1803108	64.2	1.10E-255	P0A1I3	INVA_SALTY	Invasion protein InvA
[Denovogenes]_283804	100	5.00E-136	P0AD45	QSEG_ECO57	Quorum-sensing regulator protein G
[Denovogenes]_309385	60.6	4.80E-67	P0AD45	QSEG_ECO57	Quorum-sensing regulator protein G
[Denovogenes]_994506	47.2	1.10E-24	P0AD45	QSEG_ECO57	Quorum-sensing regulator protein G

### Association between gut microbiotic structure and BA profiles

**Fig. 6:**
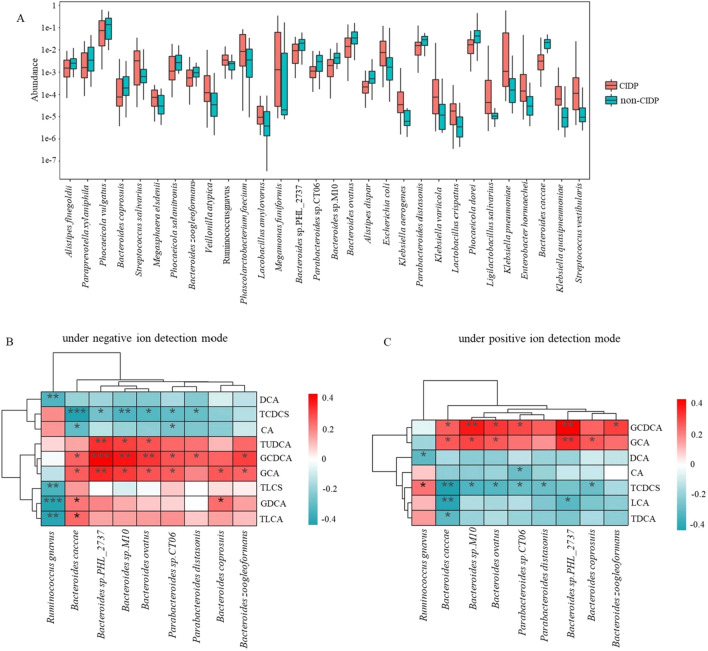
Association between the gut microbiotic abundance and BA profiles. **A** Boxplot showing bacterial species abundance in the stools of the CIDP group versus non-CIDP group. The red box shows the species with significant differences between the CIDP and non-CIDP groups. **B**, **C** Relationship between BA levels detected under the **B** negative ion mode and **C** positive ion mode and nine selected corresponding relative microbial abundances. The colored scale shows the relative abundances and correlation values. *p < 0.05, **p < 0.01, ***p < 0.0001

Secondary BAs are produced by gut microbes, such as *Bacteroides*, *Clostridium*, *Lactobacillus,* and *Ruminococcus*, via transformation of host-derived primary BAs [45]. In this study, the secondary BA profiles of CIDP subjects and non-CIDP subjects had notable differences (Table 1), indicating that the secondary BA metabolic pathways of microorganisms were abnormal in CIDP. Metagenome data revealed that gut microbes associated with the transformation of secondary BAs, including several genus of *Bacteroides*, *Parabacteroides* and *Ruminococcus*, were decreased in CIDP subjects versus non-CIDP subjects (Fig. 6A), which may result in abnormal transformation of secondary BAs. To further elucidate any correlation between these bacterial species and secondary BA transformation, Pearson’s correlation analyses were performed. Results revealed that the decreased TUDCA level in the serum of CIDP subjects significantly and positively correlated with *Bacteroides* sp. PHL_2737, *Bacteroides* sp. M10, and *Bacteroides ovatus*; the decreased GDCA level significantly and positively correlated with *Bacteroides caccae* and *Bacteroides coprosuis*; the decreased TLCA level significantly and positively correlated with *Bacteroides caccae*; and the increased TCDCS significantly and positively correlated with *Ruminococcus gnavus* (Fig. 6B, C). These results suggested that changes in the secondary BA profile might be associated with an altered composition of gut microbiota.

## Discussion

Although *Campylobacter jejuni* infection is known to be the pathophysiological basis of neural involvement leading to GBS [14, 15], surprisingly, this pathogen was not detected in any of the stool samples from CIDP subjects in our study. However, our study revealed that gut microbiome structures differed significantly between the CIDP group and non-CIDP group at the family, genus, and species levels. *Klebsiella pneumonia* (Enterobacteriaceae), *Escherichia coli* (Enterobacteriaceae), *Megamonas funiformis* (Selenomonadaceae), and *Phascolarctobacterium faecium* (Acidaminococcaceae) were the most enriched species in group CIDP, and Wilcoxon rank-sum test analysis indicated that *Klebsiella pneumoniae* was the most significantly differing species between the CIDP and non-CIDP groups in terms of abundance. Many virulence factor-encoding genes and infection-related genes were enriched in CIDP subjects (Table 2, Additional file 1: Tables S5-S6). Besides, KEGG analysis of metagenome data also revealed that genes belonging to pathways associated with bacterial invasion of epithelial cells and bacterial infection were enriched in CIDP groups (Additional file 1: Table S5 and Figure S4). *Klebsiella pneumonia* and *Escherichia coli* are well-known opportunistic pathogens*,* and *Megamonas funiformis* may be associated with inflammatory bowel disease [46], colorectal cancer [47], multiple system atrophy [48], and myasthenia gravis [49]. *Phascolarctobacterium faecium* is potentially associated with the metabolic state and mood of the host [50]. These data support a link between the gut microbial infection and CIDP, although the function of these opportunistic pathogens in CIDP remains unclear.

Currently, there are no definitive biomarkers for CIDP. In this study, the serum BA profile was notably altered in CIDP subjects versus healthy subjects. BA metabolic disorders are associated with type 2 diabetes, type 1 diabetes mellitus, dyslipidemia, non-alcoholic fatty liver disease, and some neurodegenerative disorders [51–55]. Moreover, previous studies suggest that altered BA profiles are found with cirrhosis, schizophrenia, and Alzheimer's disease and that BAs may be candidate diagnostic biomarkers for these diseases [56–58]. In our study, host-derived primary BAs (CA, TCA, and TCDCS) were increased by over eightfold in the serum of CIDP subjects, whereas microbial-derived secondary BAs (GDCA, TUDCA, TLCA, and TLCS) were decreased. Notably, BA administration, particularly that of TUDCA and ursodeoxycholic acid (UDCA), contributed to neurologic symptom improvements in animal models of Alzheimer’s, Parkinson’s, and Huntington disease [59–61]. Here, TUDCA was also noticeably decreased in CIDP subjects; however, the relationship between TUDCA and CIDP remains unclear. Additionally, other differences were found in the serum BA profile of the CIDP group, as the conjugated serum BAs to free BAs ratio was decreased, while TCA/DCA and TCDCA/LCA ratios were increased. Interestingly, dehydrocholic acid (DHCA), which was considered as synthetic BA for clinical use previously, increased by about 47-fold in CIDP versus non-CIDP (Table 1), while CIDP patients were not treated with DHCA drug. These data suggested that the levels of some specific serum BAs may change with the development of CIDP and further suggested that dysmetabolism of BAs may be used as a surrogate biomarker to distinguish CIDP patients from the healthy population. However, their predictive value still needs further investigation and validation in larger prospective cohort studies.

**Fig. 7:**
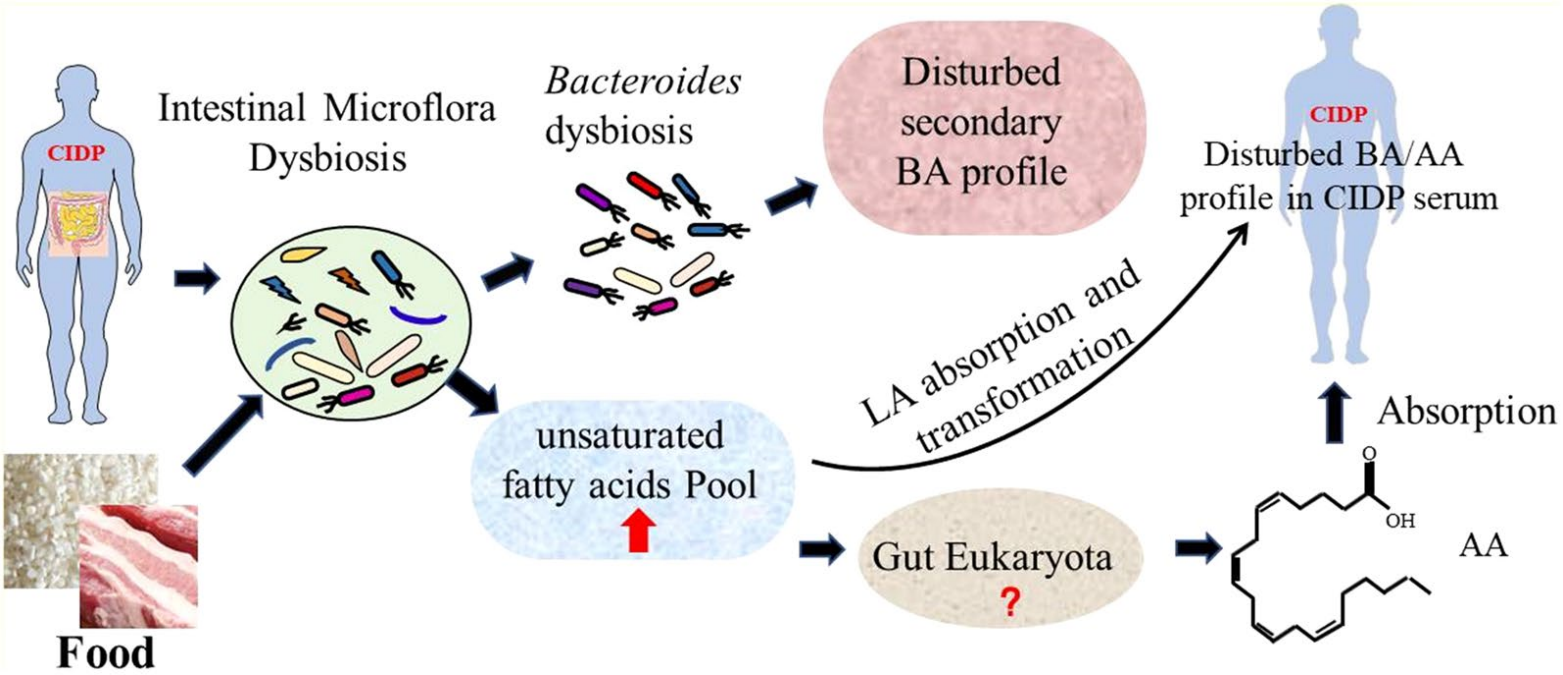
Potential routes by which intestinal microorganisms could impact serum BA/AA levels in CIDP. BA: Bile acid; LA: linoleic acid; AA: arachidonic acid

Multiple studies have focused on the effect of intestinal microorganisms on secondary BA conversion as host-derived primary BAs are deconjugated via microbial bile salt hydrolases (BSH) and then transformed into secondary BAs through microbial hydroxysteroid dehydrogenases, which are widely found in the gut microbiota, including *Bacteroides*, *Clostridium*, *Bifidobacterium*, *Eubacterium*, *Lactobacillus*, *Peptostreptococcus*, *Listeria,* and *Enterococcus* [62–65]. However, the effects of gut microbiota in AA biosynthesis are unclear, although many environmental microorganisms have been reported to produce AA, including fungi of the genera *Mortierella* [66–68], *Diasporangium* [69], and *Pythium* [70], and bacteria such as *Aureispira maritime* [71]. In this study, AA metabolism was found abnormal in CIDP subjects, and the fecal metagenome data revealed that the number of eukaryotic microbes in the host gut was slightly increased in the CIDP group (0.035%) versus non-CIDP group (0.021%), although the differences were not statistically significant (Additional file 1: Fig S5). Additionally, KEGG level 3 analyses revealed increased gene abundance associated with steroid metabolism, unsaturated fatty acid biosynthesis, and linolenic acid and glycerolipid metabolism in the gut microbes of CIDP subjects (Additional file 1: Table S5), suggesting that intestinal microbes in CIDP patients might enhance the degradation of food into short chain fatty acids, including linolenic acid. Furthermore, metabolome data showed that the levels of serum linoleic acid and 8z, 11z, 14z-eicosatrienoic acid (an intermediate of AA synthesized from linoleic acid) were increased in the CIDP group (Table 1). In summary, we speculate that the dysbiosis of gut microbiota in CIDP subjects may result in the abnormal AA content in host serum as intestinal microbes could ferment food into short chain fatty acids, including linoleic acid, to provide raw materials for intestinal microbes or the host to synthesize AA (Fig. 7). However, how eukaryotic microorganisms in the gut affect AA synthesis in CIDP subjects remains to be further elucidated.

Microbes in healthy human guts belong to a small number of families, mainly dominated by Bacteroidetes and Firmicutes [72, 73]. Here, metagenomics data revealed that the gut microbial population changed significantly in CIDP subjects. For example, the abundance of *Bacteroides* (Bacteroidaceae, Bacteroidetes) was much lower in the stool samples of the CIDP subjects than non-CIDP subjects, while pathogenic bacteria such as *Klebsiella pneumonia* (Enterobacteriaceae) were enriched in the stool samples of the CIDP subjects. KEGG annotation of metagenomic data also revealed that genes involved in bacterial infection and disease were enriched in CIDP subjects. These findings indicate that the normal microbiota-host interactions are disturbed with CIDP.

The past few years have brought exciting new insights in the field of probiotic therapies. Guo et al. reviewed the potentials and challenges of using *Clostridium* species as probiotics to impact human heath through affecting microbe-host interactions [74]. Foley et al. investigated the role of BSHs encoded by *Lactobacillus gasseri* and *Lactobacillus acidophilus*, which were used as probiotics, and revealed that BA type and BSH substrate preferences affected the growth of these two species [75]. These reports contribute to the understanding of probiotic bacterial survival mechanisms in various BA-rich niches and provide information for future use of probiotics as therapeutic tools for manipulating gut microbiota. Given that our findings suggest abnormal microbiota-host interactions are present in CIDP patients, we propose using probiotics to improve these interactions in such patients through the gut-BA-host axis. Indeed, further development of probiotics efficient in secondary BA conversion could provide an augmenting therapy for CIDP.

## Conclusion

This study revealed broad metabolic shifts in CIDP subjects versus control subjects, with perturbations in the serum BA profile and significantly increased AA in CIDP. Additionally, the gut microbiome composition was significantly changed in CIDP subjects, and levels of gut microbes related to secondary BA biotransformation were abnormal in CIDP. Our findings suggest that CIDP may be related to alterations in the gut microbiome, including potential infection with pathogens, and that the dysbiosis of gut microbial flora impacts BA and AA metabolism in CIDP subjects. Our findings also suggest that a combination of BAs and AA could be used as a surrogate biomarker of CIDP and that modulation of gut microbiota could impact the CIDP clinical course.

## Supplementary Information

**Additional file 1: Table S1.** The cohort of CIDP patients and healthy controls enrolled in this study. **Table S2.** Enriched metabolites and their pathways identified in the negative ion mode. **Table S3.** Enriched metabolites and their pathways identified in the positive ion mode. **Table S4.** Enriched genes involved in virulence factors in CIDP subjects. **Table S5.** KEGG 3 pathways with significant changes in gene abundance. **Table S6.** Enriched genes involved in secretion system in CIDP subjects. **Figure S1.** Rarefaction curve Boxplot. Abscissa represents for sample size while ordinate represents for number of species in sample. Diversity is limited when sample size is small, which is not reliable to represent for the entire microbiota structure. When rarefaction curve tends to be steady, it indicates that the sampling quantity is sufficient. **Figure S2.** NMDS and Anosim analysis. Anosim analysis at the family-level (A), genus-level (C) and the species-level (E). Between represents the distance between CIDP and non-CIDP groups, and the remaining boxes represent the distance within the corresponding group. NMDS analysis at the family-level (B), genus-level (D) and the species-level (F). Scales on X-axis and Y-axis on NMDS graph are the projection axes of samples in 2D. **Figure S3.** Microbial classifications and abundance in individual samples shown at family-level (A), genus-level (B) and the species-level (C). **Figure S4.** Box plot of enriched pathways in CIDP of KEGG secondary classification. The gene number can be viewed on the X-axis, the secondary classification can be viewed on Y-axis. **Figure S5.** Comparisons of the relative Eukaryota-Bacteria ratio in the stool of non-CIDP and CIDP group. Data are shown as mean ± SEM.

## Data Availability

Metagenome data were deposited to NCBI with SRA data: PRJNA878638. Metabolomics data have been deposited to the EMBL-EBI MetaboLights database with the identifier MTBLS5828.
