# Supplementary material for: Metabolic disorder and intestinal microflora dysbiosis in chronic inflammatory demyelinating polyradiculoneuropathy
Source: Cell Biosci. 2023 Jan 11;13:6. doi: 10.1186/s13578-023-00956-1 (PMC9832664; doi:10.1186/s13578-023-00956-1)
Supplement: Supplementary file 1 — Additional file 1: Table S1. The cohort of CIDP patients and healthy controls enrolled in this study. Table S2. Enriched metabolites and their pathways identified in the negative ion mode. Table S3. Enriched metabolites and their pathways identified in the positive ion mode. Table S4. Enriched genes involved in virulence factors in CIDP subjects. Table S5. KEGG 3 pathways with significant changes in gene abundance. Table S6. Enriched genes involved in secretion system in CIDP subjects. Figure S1. Rarefaction curve Boxplot. Abscissa represents for sample size while ordinate represents for number of species in sample. Diversity is limited when sample size is small, which is not reliable to represent for the entire microbiota structure. When rarefaction curve tends to be steady, it indicates that the sampling quantity is sufficient. Figure S2. NMDS and Anosim analysis. Anosim analysis at the family-level (A), genus-level (C) and the species-level (E). Between represents the distance between CIDP and non-CIDP groups, and the remaining boxes represent the distance within the corresponding group. NMDS analysis at the family-level (B), genus-level (D) and the species-level (F). Scales on X-axis and Y-axis on NMDS graph are the projection axes of samples in 2D. Figure S3. Microbial classifications and abundance in individual samples shown at family-level (A), genus-level (B) and the species-level (C). Figure S4. Box plot of enriched pathways in CIDP of KEGG secondary classification. The gene number can be viewed on the X-axis, the secondary classification can be viewed on Y-axis. Figure S5. Comparisons of the relative Eukaryota-Bacteria ratio in the stool of non-CIDP and CIDP group. Data are shown as mean ± SEM. [file 13578_2023_956_MOESM1_ESM.doc]

**Additional file material**

**Metabolic Disorder and Intestinal Microflora Dysbiosis in CIDP**

Jiafang Fu, PhD,^1,3,4^ Jingli Shan, PhD,^2^ Yazhou Cui, PhD,^1,3,4^ Chuanzhu Yan, PhD,^2,5,6^ Qinzhou Wang, PhD,^2^† Jinxiang Han, PhD,^1,3,4^†and Guangxiang Cao, PhD,^1,3,4^†

From the ^1^First Affiliated Hospital of Shandong First Medical University, Biomedical Sciences College & Shandong Medicinal Biotechnology Centre, Shandong First Medical University & Shandong Academy of Medical Sciences, Jinan 250117, China;

^2^Research Institute of Neuromuscular and Neurodegenerative Diseases and Department of Neurology, Qilu Hospital, Cheeloo College of Medicine, Shandong University, Jinan 250012, China;

^3^Key Lab for Rare & Uncommon Diseases of Shandong Province, Jinan 250117, China;

^4^NHC Key Laboratory of Biotechnology Drugs, Shandong Academy of Medical Sciences, Jinan 250117, China;

^5^Department of Central Laboratory and Mitochondrial Medicine Laboratory, Qilu Hospital (Qingdao), Cheeloo College of Medicine, Shandong University, Qingdao, China 266035; and

^6^Brain Science Research Institute, Shandong University, Jinan 250012, China

Address correspondence to Qinzhou Wang, Shandong University, Cheeloo College of Medicine, Jinan 250012, China. E-mail: Qinzhouwang@163.com, JInxiang Han, Shandong First Medical University & Shandong Academy of Medical Sciences, Jinan 250117, China. E-mail: jxhan@sdfmu.edu.cn, and Guangxiang Cao, Shandong First Medical University & Shandong Academy of Medical Sciences, Jinan 250117, China. E-mail: caoguangxiang@sdfmu.edu.cn.

†These authors contributed equally to the conception and supervision of this work.

Table S1 The cohort of CIDP patients and healthy controls enrolled in this study.

| **Subject ID** | **age** | **gender** | **subject** | **with cardiovascular disease** |
| --- | --- | --- | --- | --- |
| D1 | 33 | male | CIDP | No |
| D2 | 68 | male | CIDP | Yes |
| D3 | 33 | male | CIDP | No |
| D4 | 48 | female | CIDP | No |
| D5 | 44 | female | CIDP | No |
| D6 | 22 | male | CIDP | No |
| D7 | 61 | male | CIDP | Yes |
| D8 | 19 | female | CIDP | No |
| D9 | 51 | male | CIDP | Yes |
| D10 | 73 | male | CIDP | No |
| D12 | 35 | male | CIDP | No |
| D13 | 28 | male | CIDP | No |
| D14 | 55 | female | CIDP | No |
| D15 | 26 | female | CIDP | No |
| D16 | 45 | male | CIDP | No |
| D17 | 62 | female | CIDP | Yes |
| D18 | 47 | female | CIDP | Yes |
| D19 | 23 | male | CIDP | No |
| D20 | 59 | female | CIDP | No |
| D21 | 31 | female | CIDP | No |
| D22 | 51 | male | CIDP | Yes |
| D24 | 33 | male | CIDP | No |
| D25 | 58 | male | CIDP | No |
| D26 | 37 | male | CIDP | No |
| D27 | 17 | male | CIDP | No |
| D28 | 55 | female | CIDP | Yes |
| D29 | 67 | male | CIDP | No |
| D30 | 54 | male | CIDP | Yes |
| D31 | 61 | male | CIDP | No |
| D32 | 33 | female | CIDP | No |
| D33 | 49 | female | CIDP | No |
| K1 | 35 | male | non-CIDP | No |
| K2 | 66 | male | non-CIDP | No |
| K3 | 33 | male | non-CIDP | No |
| K4 | 48 | female | non-CIDP | No |
| K5 | 40 | female | non-CIDP | No |
| K6 | 23 | male | non-CIDP | No |
| K7 | 61 | male | non-CIDP | No |
| K8 | 20 | female | non-CIDP | No |
| K9 | 50 | male | non-CIDP | No |
| K10 | 73 | male | non-CIDP | No |
| K11 | 34 | male | non-CIDP | No |
| K12 | 29 | male | non-CIDP | No |
| K13 | 55 | female | non-CIDP | No |
| K14 | 25 | female | non-CIDP | No |
| K15 | 44 | male | non-CIDP | No |
| K16 | 63 | female | non-CIDP | No |
| K17 | 47 | female | non-CIDP | No |
| K18 | 22 | male | non-CIDP | No |
| K19 | 57 | female | non-CIDP | No |
| K20 | 31 | female | non-CIDP | No |
| K21 | 51 | male | non-CIDP | No |
| K22 | 30 | male | non-CIDP | No |
| K23 | 56 | male | non-CIDP | No |
| K24 | 37 | male | non-CIDP | No |
| K25 | 17 | male | non-CIDP | No |
| K26 | 55 | female | non-CIDP | No |
| K27 | 69 | male | non-CIDP | No |
| K28 | 56 | male | non-CIDP | No |
| K29 | 62 | male | non-CIDP | No |
| K30 | 30 | female | non-CIDP | No |
| K31 | 47 | female | non-CIDP | No |
| K32 | 22 | male | non-CIDP | No |
| K33 | 31 | female | non-CIDP | No |

Table S2 Enriched metabolites and their pathways identified in the negative ion mode

| Pathway | Number of DA metabolites | Total number of metabolites in the pathway | P-value | DA metabolites |
| --- | --- | --- | --- | --- |
| Caffeine metabolism | 4 | 22 | 3.43626E-06 | Xanthine; 7-methylxanthine; Theophylline; Xanthosine |
| Metabolic pathways | 21 | 1706 | 4.57323E-05 | Taurine;L-(+)-lactic acid; Hypoxanthine; Xanthine; Inosine; 7-methylxanthine; Homogentisic acid; 4-methylcatechol; 4-hydroxyphenylpyruvic acid; Theophylline; Oxalosuccinic acid; 3-hydroxyanthranilic acid; Caprylic acid; Thromboxane b2;4-coumaroylshikimic acid; Xanthosine; Cysteinylglycine; L-cysteine; Glycocholic acid; Cholic acid; Arachidonic acid |
| **Primary bile acid biosynthesis** | 4 | 47 | 7.64354E-05 | Taurine;Glycocholic acid;Cholic acid;Glycochenodeoxycholate |
| Cholesterol metabolism | 2 | 10 | 0.001005813 | Glycocholic acid; Glycochenodeoxycholate |
| Purine metabolism | 4 | 95 | 0.001144564 | Hypoxanthine; Xanthine; Inosine; Xanthosine |
| **Bile secretion** | 4 | 97 | 0.001236644 | Thromboxane b2; Glycocholic acid; Cholic acid; Glycochenodeoxycholate |
| Taurine and hypotaurine metabolism | 2 | 22 | 0.004972978 | Taurine; L-cysteine |
| **Linoleic acid metabolism** | 2 | 28 | 0.0079868 | (+/-)12(13)-dihome; Arachidonic acid |
| Ferroptosis | 2 | 29 | 0.008551747 | L-cysteine; Arachidonic acid |
| Sulfur metabolism | 2 | 33 | 0.01098396 | Taurine; L-cysteine |
| Central carbon metabolism in cancer | 2 | 37 | 0.01368369 | L-(+)-lactic acid; L-cysteine |
| Glutathione metabolism | 2 | 38 | 0.01439914 | Cysteinylglycine; L-cysteine |
| Serotonergic synapse | 2 | 42 | 0.017418 | Thromboxane b2; Arachidonic acid |
| GnRH signaling pathway | 1 | 6 | 0.02854508 | Arachidonic acid |
| Leishmaniasis | 1 | 6 | 0.02854508 | Arachidonic acid |
| Phenylalanine metabolism | 2 | 60 | 0.03387121 | N-phenylacetylglutamine; 3-phenyllactic acid |
| Fc gamma R-mediated phagocytosis | 1 | 8 | 0.03787989 | Arachidonic acid |
| Long-term depression | 1 | 9 | 0.04251397 | Arachidonic acid |
| Necroptosis | 1 | 10 | 0.04712599 | Arachidonic acid |
| **Biosynthesis of unsaturated fatty acids** | 2 | 74 | 0.04951326 | 11(z),14(z)-eicosadienoic acid; Arachidonic acid |

DA: differentially abundant

Table S3 Enriched metabolites and their pathways identified in the positive ion mode

| **X.Pathway** | **Number of DA metabolites** | **Total number of metabolites in the pathway** | **P-value** | **DA metabolites** |
| --- | --- | --- | --- | --- |
| Caffeine metabolism | 7 | 22 | 6.79072E-11 | Xanthine; 7-methylxanthine; 3-methylxanthine; Theobromine; Paraxanthine; Theophylline; Caffeine |
| Metabolic pathways | 28 | 1706 | 4.0011E-06 | Hypoxanthine; Xanthine; Adenosine; Guanine; Inosine; 7-methylxanthine; 3-methylxanthine; Theobromine; Paraxanthine; Theophylline; Phthalic acid; Caffeine; Pyridoxal 5'-phosphate; 3-deoxy-d-manno-octulosonic acid; Cortisone; Desoxycortone; Cortisol; Ajmalicine; Corticosterone; Protocatechuic acid; Bilirubin; Cholate; Phenol, 2-(3,7-dimethylocta-2,6-dienyl)-; Homogentisate; Thromboxane b2; D-erythro-sphingosine 1-phosphate; Bisphenol a; 6alpha-hydroxy-castasterone |
| Purine metabolism | 5 | 95 | 0.000400502 | Hypoxanthine; Xanthine; Adenosine; Guanine; Inosine |
| **Bile secretion** | 5 | 97 | 0.000440869 | Cortisol; Bilirubin; Cholate; Glycochenodeoxycholate; Thromboxane b2 |
| Steroid hormone biosynthesis | 5 | 99 | 0.000484231 | Cortisone; Desoxycortone; Cortisol; Tetrahydrocortisone; Corticosterone |
| Aldosterone-regulated sodium reabsorption | 2 | 8 | 0.001159493 | Cortisone; Cortisol |
| Prostate cancer | 2 | 11 | 0.002248477 | Cortisone; Cortisol |
| Regulation of lipolysis in adipocytes | 2 | 14 | 0.00367275 | Adenosine; Corticosterone |
| Sphingolipid signaling pathway | 2 | 15 | 0.004219709 | Adenosine; D-erythro-sphingosine 1-phosphate |
| Neuroactive ligand-receptor interaction | 3 | 52 | 0.004786366 | Adenosine; Cortisol; D-erythro-sphingosine 1-phosphate |
| Aldosterone synthesis and secretion | 2 | 22 | 0.009010203 | Desoxycortone; Corticosterone |
| Pathways in cancer | 2 | 31 | 0.01745692 | Cortisone; Cortisol |
| Prion diseases | 1 | 3 | 0.01950928 | Corticosterone |
| **Primary bile acid biosynthesis** | 2 | 47 | 0.0379322 | Cholate; Glycochenodeoxycholate |

DA: differentially abundant

Table S4 Enriched genes involved in virulence factors in CIDP subjects.

| Gene_id | Identity | E_value | gene | protein | description |
| --- | --- | --- | --- | --- | --- |
| [denovogenes]_316146 | 92.8 | 6.70E-114 | P0DM78 | PHOP_SALTY | Virulence transcriptional regulatory protein PhoP |
| [denovogenes]_323957 | 93.3 | 5.10E-114 | E1WFA1 | PHOP_SALTS | Virulence transcriptional regulatory protein PhoP |
| [denovogenes]_1634 | 54.1 | 0.00E+00 | P25927 | BIGA_SALTY | Putative surface-exposed virulence protein BigA |
| [denovogenes]_2027 | 42.3 | 7.50E-297 | P25927 | BIGA_SALTY | Putative surface-exposed virulence protein BigA |
| [denovogenes]_592521 | 40.3 | 1.10E-06 | P24421 | VSDF_SALDU | Virulence protein VsdF |
| [denovogenes]_684238 | 46.8 | 3.40E-26 | Q8ZM36 | VIR17_SALTY | Virulence protein STM3117 |
| [denovogenes]_697909 | 42.4 | 6.30E-17 | Q8ZM36 | VIR17_SALTY | Virulence protein STM3117 |
| [denovogenes]_742681 | 46.4 | 4.10E-26 | Q8ZM36 | VIR17_SALTY | Virulence protein STM3117 |
| [denovogenes]_746965 | 44.2 | 7.80E-25 | Q8ZM36 | VIR17_SALTY | Virulence protein STM3117 |
| [denovogenes]_823719 | 54.4 | 3.40E-06 | Q46558 | VAPB_DICNO | Virulence-associated protein B |
| [denovogenes]_829120 | 94.4 | 4.30E-62 | Q57QC3 | PHOP_SALCH | Virulence transcriptional regulatory protein PhoP |
| [denovogenes]_835558 | 50.8 | 1.10E-25 | Q8ZM36 | VIR17_SALTY | Virulence protein STM3117 |
| [denovogenes]_838871 | 50 | 1.40E-25 | Q8ZM36 | VIR17_SALTY | Virulence protein STM3117 |
| [denovogenes]_906939 | 41.1 | 2.20E-07 | Q46561 | VAPZ_DICNO | Virulence-associated protein A' |
| [denovogenes]_911873 | 42.5 | 5.60E-11 | Q46560 | VAPI_DICNO | Virulence-associated protein I |
| [denovogenes]_924946 | 40.7 | 4.60E-13 | P24421 | VSDF_SALDU | Virulence protein VsdF |
| [denovogenes]_936488 | 49.3 | 5.00E-12 | Q46560 | VAPI_DICNO | Virulence-associated protein I |
| [denovogenes]_941521 | 45 | 7.10E-11 | Q46561 | VAPZ_DICNO | Virulence-associated protein A' |
| [denovogenes]_945845 | 42 | 4.20E-11 | Q46561 | VAPZ_DICNO | Virulence-associated protein A' |
| [denovogenes]_966264 | 43.2 | 2.20E-12 | Q46560 | VAPI_DICNO | Virulence-associated protein I |
| [denovogenes]_990501 | 42.9 | 1.40E-08 | Q46560 | VAPI_DICNO | Virulence-associated protein I |
| [denovogenes]_992669 | 40.3 | 6.40E-09 | Q46560 | VAPI_DICNO | Virulence-associated protein I |
| [denovogenes]_999748 | 44.9 | 8.10E-12 | Q46560 | VAPI_DICNO | Virulence-associated protein I |
| [denovogenes]_1023098 | 49.3 | 1.60E-12 | Q46560 | VAPI_DICNO | Virulence-associated protein I |
| [denovogenes]_1025202 | 55.7 | 5.20E-19 | Q46560 | VAPI_DICNO | Virulence-associated protein I |
| [denovogenes]_1060369 | 43.8 | 1.50E-07 | Q46560 | VAPI_DICNO | Virulence-associated protein I |
| [denovogenes]_1065039 | 40.8 | 9.80E-07 | Q46560 | VAPI_DICNO | Virulence-associated protein I |
| [denovogenes]_1065726 | 49.3 | 2.90E-11 | Q46560 | VAPI_DICNO | Virulence-associated protein I |
| [denovogenes]_1078776 | 40.8 | 6.70E-08 | Q46560 | VAPI_DICNO | Virulence-associated protein I |
| [denovogenes]_1082003 | 41.1 | 4.50E-12 | Q46560 | VAPI_DICNO | Virulence-associated protein I |
| [denovogenes]_1091528 | 45.6 | 2.90E-11 | Q46560 | VAPI_DICNO | Virulence-associated protein I |
| [denovogenes]_1094893 | 41 | 5.80E-12 | Q46560 | VAPI_DICNO | Virulence-associated protein I |
| [denovogenes]_1114270 | 52.2 | 2.40E-18 | Q46560 | VAPI_DICNO | Virulence-associated protein I |
| [denovogenes]_1145114 | 97.3 | 4.70E-35 | Q05459 | VAGC_SALDU | Virulence-associated protein VagC |
| [denovogenes]_1150395 | 54.5 | 7.60E-17 | Q46560 | VAPI_DICNO | Virulence-associated protein I |
| [denovogenes]_1163988 | 93.4 | 5.20E-34 | Q05459 | VAGC_SALDU | Virulence-associated protein VagC |
| [denovogenes]_1171642 | 41.3 | 5.90E-14 | Q46560 | VAPI_DICNO | Virulence-associated protein I |
| [denovogenes]_1173346 | 41.1 | 1.70E-13 | Q46560 | VAPI_DICNO | Virulence-associated protein I |
| [denovogenes]_1179973 | 46.2 | 5.00E-13 | Q46560 | VAPI_DICNO | Virulence-associated protein I |
| [denovogenes]_1183997 | 55.6 | 2.90E-21 | Q46560 | VAPI_DICNO | Virulence-associated protein I |
| [denovogenes]_1198573 | 45.1 | 8.40E-13 | Q46560 | VAPI_DICNO | Virulence-associated protein I |
| [denovogenes]_1206473 | 50.7 | 2.40E-12 | Q46560 | VAPI_DICNO | Virulence-associated protein I |
| [denovogenes]_1250329 | 54.4 | 1.90E-06 | Q46558 | VAPB_DICNO | Virulence-associated protein B |
| [denovogenes]_1328069 | 54.5 | 6.70E-19 | P24421 | VSDF_SALDU | Virulence protein VsdF |
| [denovogenes]_1353024 | 65 | 3.30E-23 | P0A1G4 | MSGA_SALTI | Virulence protein MsgA |
| [denovogenes]_1365834 | 77.2 | 8.00E-30 | P0A1G3 | MSGA_SALTY | Virulence protein MsgA |
| [denovogenes]_1384852 | 97.4 | 7.30E-36 | Q05459 | VAGC_SALDU | Virulence-associated protein VagC |
| [denovogenes]_1392203 | 46.7 | 6.50E-13 | Q46558 | VAPB_DICNO | Virulence-associated protein B |
| [denovogenes]_1392236 | 48.7 | 9.40E-12 | Q46558 | VAPB_DICNO | Virulence-associated protein B |
| [denovogenes]_1392445 | 50 | 1.30E-13 | Q46558 | VAPB_DICNO | Virulence-associated protein B |
| [denovogenes]_1392520 | 44.6 | 5.20E-10 | Q46558 | VAPB_DICNO | Virulence-associated protein B |
| [denovogenes]_1392702 | 81.6 | 1.20E-30 | Q05459 | VAGC_SALDU | Virulence-associated protein VagC |
| [denovogenes]_1392777 | 97.4 | 1.60E-35 | Q05459 | VAGC_SALDU | Virulence-associated protein VagC |
| [denovogenes]_1393358 | 48.6 | 4.20E-12 | Q46558 | VAPB_DICNO | Virulence-associated protein B |
| [denovogenes]_1393779 | 48.6 | 2.00E-14 | Q46558 | VAPB_DICNO | Virulence-associated protein B |
| [denovogenes]_1393904 | 45.9 | 1.80E-10 | Q46558 | VAPB_DICNO | Virulence-associated protein B |
| [denovogenes]_1394087 | 49.3 | 3.50E-14 | Q46558 | VAPB_DICNO | Virulence-associated protein B |
| [denovogenes]_1394518 | 48.6 | 3.50E-14 | Q46558 | VAPB_DICNO | Virulence-associated protein B |
| [denovogenes]_1395443 | 96 | 2.10E-35 | Q05459 | VAGC_SALDU | Virulence-associated protein VagC |
| [denovogenes]_1395475 | 94.7 | 7.90E-35 | Q05459 | VAGC_SALDU | Virulence-associated protein VagC |
| [denovogenes]_1397988 | 46.7 | 3.20E-12 | Q46558 | VAPB_DICNO | Virulence-associated protein B |
| [denovogenes]_1399613 | 97.4 | 9.40E-36 | Q05459 | VAGC_SALDU | Virulence-associated protein VagC |
| [denovogenes]_1401055 | 52.7 | 1.20E-14 | Q46558 | VAPB_DICNO | Virulence-associated protein B |
| [denovogenes]_1405374 | 47.2 | 6.00E-11 | Q46558 | VAPB_DICNO | Virulence-associated protein B |
| [denovogenes]_1417313 | 52.7 | 3.40E-14 | Q46558 | VAPB_DICNO | Virulence-associated protein B |
| [denovogenes]_1521014 | 51.9 | 1.70E-09 | Q46560 | VAPI_DICNO | Virulence-associated protein I |
| [denovogenes]_1881219 | 79.3 | 6.30E-226 | Q57QC4 | PHOQ_SALCH | Virulence sensor histidine kinase PhoQ |
| [denovogenes]_90570 | 42.7 | 1.30E-57 | P0A247 | VIRB_SHIFL | Virulence regulon transcriptional activator VirB |
| [denovogenes]_93514 | 42.6 | 1.70E-54 | P0A248 | VIRB_SHISO | Virulence regulon transcriptional activator VirB |
| [denovogenes]_724004 | 47.6 | 5.00E-19 | P26316 | HRPS_PSESH | Pathogenicity locus probable regulatory protein HrpS |

Table S5 KEGG 3 pathways with significant changes in gene abundance

| **KEGG 3 pathway** | **p-value** | **median** | | **rank.mean** | | **rank.median** | |
| --- | --- | --- | --- | --- | --- | --- | --- |
|  |  | **CIDP** | **non-CIDP** | **CIDP** | **non-CIDP** | **CIDP** | **non-CIDP** |
| **Pathways associated with infection** | | | | | | | |
| Bacterial invasion of epithelial cells | 0.000261689 | 2.262965 | 0.366621 | 39.7857 | 23.5455 | 41.5 | 21 |
| Pathogenic *Escherichia coli* infection | 0.002268697 | 131.5015 | 119.9852 | 38.4286 | 24.697 | 42.5 | 23 |
| *Yersinia* infection | 0.030464291 | 0.352572 | 0.08987 | 36.3571 | 26.4545 | 41.5 | 26 |
| *Staphylococcus aureus* infection | 7.05E-05 | 19.07688 | 7.867943 | 40.5 | 22.9394 | 42.5 | 20 |
| Quorum sensing | 0.000335063 | 4232.143 | 3706.929 | 39.6429 | 23.6667 | 42.5 | 20 |
| Bacterial secretion system | 0.000105897 | 2543.647 | 2215.483 | 40.2857 | 23.1212 | 43 | 20 |
| **Pathways might be associated with bile acids and arachidonic acid** | | | | | | | |
| Steroid degradation | 0.001213031 | 0.046431 | 0 | 38.6071 | 24.5455 | 43.5 | 14.5 |
| Biosynthesis of unsaturated fatty acids | 0.013405892 | 11.44181 | 8.345596 | 37.0714 | 25.8485 | 39.5 | 24 |
| Alpha Linolenic acid metabolism | 0.017905514 | 6.999126 | 3.522634 | 36.8214 | 26.0606 | 43.5 | 26 |
| Glycerolipid_metabolism | 0.006327964 | 947.3545 | 810.7326 | 37.6786 | 25.3333 | 40.5 | 22 |
| Carbon metabolism | 0.037161596 | 8095.555 | 7582.574 | 36.1429 | 26.6364 | 42 | 25 |

Table S6 Enriched genes involved in secretion system in CIDP subjects.

| Gene_id | Identity | E_value | gene | protein | description |
| --- | --- | --- | --- | --- | --- |
| [denovogenes]_191047 | 58.5 | 8.50E-79 | P15643 | GSPC_KLEPN | Type II secretion system protein C |
| [denovogenes]_191785 | 57.8 | 6.10E-77 | P15643 | GSPC_KLEPN | Type II secretion system protein C |
| [denovogenes]_191786 | 58.4 | 3.60E-77 | P15643 | GSPC_KLEPN | Type II secretion system protein C |
| [denovogenes]_191937 | 44.3 | 1.90E-30 | Q9RPY4 | VIRB1_BRUSU | Type IV secretion system protein virB1 |
| [denovogenes]_199025 | 99.6 | 3.70E-151 | P45757 | GSPC_ECOLI | Type II secretion system protein C |
| [denovogenes]_199760 | 44.4 | 1.80E-60 | Q9I741 | VGR1A_PSEAE | Type VI secretion system spike protein VgrG1a |
| [denovogenes]_200295 | 54.9 | 2.20E-71 | Q2YJ82 | VIRBB_BRUA2 | Type IV secretion system protein VirB11 |
| [denovogenes]_200912 | 52.2 | 4.00E-81 | P31741 | GSPE_AERHY | Type II secretion system protein E |
| [denovogenes]_202061 | 47 | 2.90E-63 | P37093 | GSPE_VIBCH | Type II secretion system ATPase E |
| [denovogenes]_204912 | 40.5 | 4.50E-24 | Q7VSX3 | PTLH_BORPE | Type IV secretion system protein PtlH |
| [denovogenes]_213501 | 40.3 | 8.90E-25 | Q7W2T7 | PTLH_BORPA | Type IV secretion system protein PtlH homolog |
| [denovogenes]_220630 | 45.5 | 2.60E-32 | Q9RPY4 | VIRB1_BRUSU | Type IV secretion system protein virB1 |
| [denovogenes]_227197 | 63.1 | 4.90E-84 | P40290 | SCTN_YEREN | Type 3 secretion system ATPase |
| [denovogenes]_230761 | 48.1 | 2.20E-60 | P45759 | GSPE_ECOLI | Type II secretion system protein E |
| [denovogenes]_240652 | 51.6 | 6.10E-63 | P15645 | GSPE_KLEPN | Type II secretion system protein E |
| [denovogenes]_254822 | 48.4 | 9.40E-61 | P15753 | GSPN_KLEPN | Type II secretion system protein N |
| [denovogenes]_256671 | 49.2 | 4.50E-63 | P15753 | GSPN_KLEPN | Type II secretion system protein N |
| [denovogenes]_262027 | 41.8 | 1.80E-24 | Q2YIT5 | VIRB1_BRUA2 | Type IV secretion system protein virB1 |
| [denovogenes]_266389 | 41.2 | 3.40E-47 | Q2YJ78 | VIRB8_BRUA2 | Type IV secretion system protein virB8 |
| [denovogenes]_271364 | 50.4 | 9.80E-63 | P15753 | GSPN_KLEPN | Type II secretion system protein N |
| [denovogenes]_280170 | 56.7 | 3.80E-67 | P15751 | GSPL_KLEPN | Type II secretion system protein L |
| [denovogenes]_287568 | 41.4 | 3.80E-43 | Q7CEG3 | VIRB8_BRUSU | Type IV secretion system protein virB8 |
| [denovogenes]_290513 | 49.2 | 8.50E-27 | Q9RPY4 | VIRB1_BRUSU | Type IV secretion system protein virB1 |
| [denovogenes]_292123 | 45.4 | 1.10E-29 | Q9RPY4 | VIRB1_BRUSU | Type IV secretion system protein virB1 |
| [denovogenes]_298144 | 47.4 | 8.10E-46 | Q9I5N9 | HXCR_PSEAE | Type II secretion system protein HxcR |
| [denovogenes]_301078 | 45.4 | 9.50E-31 | Q9RPY4 | VIRB1_BRUSU | Type IV secretion system protein virB1 |
| [denovogenes]_311172 | 44.2 | 2.00E-28 | Q9RPY4 | VIRB1_BRUSU | Type IV secretion system protein virB1 |
| [denovogenes]_311209 | 40.6 | 1.10E-42 | Q9I5N9 | HXCR_PSEAE | Type II secretion system protein HxcR |
| [denovogenes]_313194 | 99.4 | 5.90E-94 | B7MAM0 | SECM_ECO45 | Secretion monitor |
| [denovogenes]_318059 | 40.8 | 2.90E-24 | Q9RPY4 | VIRB1_BRUSU | Type IV secretion system protein virB1 |
| [denovogenes]_320699 | 42.3 | 1.00E-21 | Q2YIT5 | VIRB1_BRUA2 | Type IV secretion system protein virB1 |
| [denovogenes]_329146 | 44.5 | 1.30E-40 | P31702 | GSPE_DICCH | Type II secretion system protein E |
| [denovogenes]_368017 | 41.7 | 3.60E-37 | Q00512 | GSPE_PSEAE | Type II secretion system protein E |
| [denovogenes]_376781 | 58.3 | 7.20E-62 | P31703 | GSPE_PECCC | Type II secretion system protein E |
| [denovogenes]_383161 | 47 | 4.40E-48 | P45759 | GSPE_ECOLI | Type II secretion system protein E |
| [denovogenes]_388212 | 53.3 | 5.40E-54 | Q7ARI8 | SCTN_YERPE | Type 3 secretion system ATPase |
| [denovogenes]_404703 | 100 | 4.20E-104 | P45761 | GSPJ_ECOLI | Type II secretion system protein J |
| [denovogenes]_405215 | 70.3 | 3.30E-72 | P15749 | GSPJ_KLEPN | Type II secretion system protein J |
| [denovogenes]_407705 | 71.2 | 3.50E-74 | P15749 | GSPJ_KLEPN | Type II secretion system protein J |
| [denovogenes]_413660 | 72.1 | 9.10E-67 | P15745 | GSPF_KLEPN | Type II secretion system protein F |
| [denovogenes]_422184 | 46.7 | 9.10E-43 | Q9I5N9 | HXCR_PSEAE | Type II secretion system protein HxcR |
| [denovogenes]_423794 | 60.9 | 4.80E-52 | P20725 | GSPB_KLEPN | General secretion pathway protein B |
| [denovogenes]_429464 | 50 | 4.20E-24 | Q9RPY4 | VIRB1_BRUSU | Type IV secretion system protein virB1 |
| [denovogenes]_437512 | 78.9 | 2.70E-79 | P40290 | SCTN_YEREN | Type 3 secretion system ATPase |
| [denovogenes]_441348 | 96.8 | 6.70E-99 | A0A4C3GMC1 | GSPJ_ECOLX | Type II secretion system protein J |
| [denovogenes]_448430 | 43.5 | 7.40E-26 | Q9I745 | TSSE1_PSEAE | Type VI secretion system component TssE1 |
| [denovogenes]_482081 | 69.6 | 5.60E-63 | A8ALJ9 | SECM_CITK8 | Secretion monitor |
| [denovogenes]_485223 | 100 | 2.20E-96 | Q46832 | YGHD_ECOLI | Type II secretion system M-type protein YghD |
| [denovogenes]_486502 | 73.2 | 5.00E-64 | Q9I749 | TSSB1_PSEAE | Type VI secretion system sheath protein TssB1 |
| [denovogenes]_487524 | 92.7 | 7.70E-89 | Q46832 | YGHD_ECOLI | Type II secretion system M-type protein YghD |
| [denovogenes]_494096 | 59.1 | 3.40E-52 | P74857 | SCTN2_SALTY | SPI-2 type 3 secretion system ATPase |
| [denovogenes]_499373 | 42 | 1.00E-24 | Q9I745 | TSSE1_PSEAE | Type VI secretion system component TssE1 |
| [denovogenes]_514542 | 54.6 | 5.40E-47 | P15751 | GSPL_KLEPN | Type II secretion system protein L |
| [denovogenes]_517622 | 59.3 | 3.20E-52 | P15747 | GSPH_KLEPN | Type II secretion system protein H |
| [denovogenes]_519201 | 61.1 | 3.80E-53 | P15747 | GSPH_KLEPN | Type II secretion system protein H |
| [denovogenes]_525332 | 100 | 1.40E-92 | P41443 | GSPH_ECOLI | Type II secretion system protein H |
| [denovogenes]_534768 | 99.4 | 1.50E-89 | A6T4P0 | SECM_KLEP7 | Secretion monitor |
| [denovogenes]_538147 | 93.9 | 1.10E-84 | A6T4P0 | SECM_KLEP7 | Secretion monitor |
| [denovogenes]_3369 | 42.6 | 2.10E-266 | Q8NYF3 | ESSC_STAAW | Type VII secretion system protein EssC |
| [denovogenes]_579814 | 41.2 | 2.00E-168 | Q9RPY1 | VIRB4_BRUSU | Type IV secretion system protein virB4 |
| [denovogenes]_579855 | 40.7 | 5.90E-168 | Q8YDZ4 | VIRB4_BRUME | Type IV secretion system protein virB4 |
| [denovogenes]_580000 | 41.1 | 6.30E-170 | Q8YDZ4 | VIRB4_BRUME | Type IV secretion system protein virB4 |
| [denovogenes]_581096 | 40.3 | 1.30E-07 | P41442 | GSPG_ECOLI | Type II secretion system core protein G |
| [denovogenes]_581231 | 55.6 | 4.30E-46 | P15752 | GSPM_KLEPN | Type II secretion system protein M |
| [denovogenes]_582900 | 57.5 | 4.60E-48 | P15752 | GSPM_KLEPN | Type II secretion system protein M |
| [denovogenes]_594799 | 42 | 1.00E-07 | P31586 | GSPG_PECCC | Type II secretion system core protein G |
| [denovogenes]_597864 | 40.3 | 6.30E-18 | Q9I749 | TSSB1_PSEAE | Type VI secretion system sheath protein TssB1 |
| [denovogenes]_605043 | 71.3 | 6.80E-57 | Q9I749 | TSSB1_PSEAE | Type VI secretion system sheath protein TssB1 |
| [denovogenes]_611300 | 43.1 | 2.20E-07 | A0A0H3HDD6 | GSPG_KLEOK | Type II secretion system core protein G |
| [denovogenes]_620809 | 40.3 | 5.40E-06 | A0A0H3HDD6 | GSPG_KLEOK | Type II secretion system core protein G |
| [denovogenes]_626401 | 100 | 1.40E-83 | P36678 | GSPM_ECOLI | Type II secretion system protein M |
| [denovogenes]_631023 | 47.3 | 2.40E-30 | P26609 | FLIS_SALTY | Flagellar secretion chaperone FliS |
| [denovogenes]_640988 | 40.3 | 5.30E-06 | A0A0H3HDD6 | GSPG_KLEOK | Type II secretion system core protein G |
| [denovogenes]_641368 | 76.7 | 4.00E-62 | P45773 | GSPG_VIBCH | Type II secretion system core protein G |
| [denovogenes]_648474 | 51.1 | 8.30E-36 | Q00514 | GSPG_PSEAE | Type II secretion system core protein G |
| [denovogenes]_655904 | 75.4 | 1.40E-56 | P31586 | GSPG_PECCC | Type II secretion system core protein G |
| [denovogenes]_662720 | 41 | 5.20E-22 | Q9I745 | TSSE1_PSEAE | Type VI secretion system component TssE1 |
| [denovogenes]_677205 | 100 | 1.50E-79 | P41442 | GSPG_ECOLI | Type II secretion system core protein G |
| [denovogenes]_684029 | 90 | 2.60E-71 | P15746 | GSPG_KLEPN | Type II secretion system core protein G |
| [denovogenes]_696022 | 64.8 | 5.80E-47 | P20725 | GSPB_KLEPN | General secretion pathway protein B |
| [denovogenes]_700213 | 93 | 1.40E-69 | A6T4P0 | SECM_KLEP7 | Secretion monitor |
| [denovogenes]_700497 | 96.5 | 6.80E-72 | A6T4P0 | SECM_KLEP7 | Secretion monitor |
| [denovogenes]_700921 | 41.5 | 1.70E-06 | P45773 | GSPG_VIBCH | Type II secretion system core protein G |
| [denovogenes]_717094 | 96.4 | 1.10E-71 | P03825 | GSPB_ECOLI | General secretion pathway protein B |
| [denovogenes]_737323 | 99.3 | 4.90E-67 | P26608 | FLIS_ECOLI | Flagellar secretion chaperone FliS |
| [denovogenes]_737727 | 42.6 | 2.80E-22 | P39739 | FLIS_BACSU | Flagellar secretion chaperone FliS |
| [denovogenes]_745010 | 73.5 | 1.90E-47 | P26608 | FLIS_ECOLI | Flagellar secretion chaperone FliS |
| [denovogenes]_745071 | 70.1 | 2.20E-43 | P26609 | FLIS_SALTY | Flagellar secretion chaperone FliS |
| [denovogenes]_752603 | 42.9 | 5.00E-24 | P39739 | FLIS_BACSU | Flagellar secretion chaperone FliS |
| [denovogenes]_768138 | 40.2 | 8.20E-19 | P39739 | FLIS_BACSU | Flagellar secretion chaperone FliS |
| [denovogenes]_768476 | 72.6 | 2.50E-44 | P20440 | PULS_KLEPN | Pullulanase secretion protein PulS |
| [denovogenes]_775148 | 42.2 | 8.70E-21 | P39739 | FLIS_BACSU | Flagellar secretion chaperone FliS |
| [denovogenes]_785087 | 41.2 | 1.90E-20 | P39739 | FLIS_BACSU | Flagellar secretion chaperone FliS |
| [denovogenes]_789140 | 74.2 | 1.70E-45 | P20440 | PULS_KLEPN | Pullulanase secretion protein PulS |
| [denovogenes]_792472 | 40.3 | 3.60E-19 | P39739 | FLIS_BACSU | Flagellar secretion chaperone FliS |
| [denovogenes]_800309 | 44.2 | 9.10E-23 | P39739 | FLIS_BACSU | Flagellar secretion chaperone FliS |
| [denovogenes]_808181 | 42.5 | 2.40E-20 | P39739 | FLIS_BACSU | Flagellar secretion chaperone FliS |
| [denovogenes]_808252 | 41.9 | 1.50E-22 | P39739 | FLIS_BACSU | Flagellar secretion chaperone FliS |
| [denovogenes]_809187 | 41.9 | 9.30E-20 | P39739 | FLIS_BACSU | Flagellar secretion chaperone FliS |
| [denovogenes]_809393 | 40.3 | 7.60E-22 | P39739 | FLIS_BACSU | Flagellar secretion chaperone FliS |
| [denovogenes]_812197 | 41.1 | 2.20E-21 | P39739 | FLIS_BACSU | Flagellar secretion chaperone FliS |
| [denovogenes]_814979 | 41.1 | 1.70E-21 | P39739 | FLIS_BACSU | Flagellar secretion chaperone FliS |
| [denovogenes]_816511 | 40.3 | 2.60E-22 | P39739 | FLIS_BACSU | Flagellar secretion chaperone FliS |
| [denovogenes]_828939 | 42.4 | 1.30E-13 | P45775 | GSPI_VIBCH | Type II secretion system protein I |
| [denovogenes]_835913 | 100 | 1.60E-64 | P45760 | GSPI_ECOLI | Type II secretion system protein I |
| [denovogenes]_843220 | 43.6 | 3.90E-23 | P39739 | FLIS_BACSU | Flagellar secretion chaperone FliS |
| [denovogenes]_843918 | 42.7 | 2.60E-22 | P39739 | FLIS_BACSU | Flagellar secretion chaperone FliS |
| [denovogenes]_848312 | 74.6 | 6.20E-45 | P20440 | PULS_KLEPN | Pullulanase secretion protein PulS |
| [denovogenes]_848701 | 41.1 | 2.00E-19 | P39739 | FLIS_BACSU | Flagellar secretion chaperone FliS |
| [denovogenes]_860575 | 94.3 | 1.30E-58 | Q8VPC3 | GSPI_ECOLX | Type II secretion system protein I |
| [denovogenes]_864006 | 92.7 | 3.70E-58 | Q46832 | YGHD_ECOLI | Type II secretion system M-type protein YghD |
| [denovogenes]_868365 | 63.4 | 2.30E-36 | P20725 | GSPB_KLEPN | General secretion pathway protein B |
| [denovogenes]_874354 | 95.9 | 5.20E-60 | Q8VPC3 | GSPI_ECOLX | Type II secretion system protein I |
| [denovogenes]_875588 | 81 | 7.40E-51 | P15748 | GSPI_KLEPN | Type II secretion system protein I |
| [denovogenes]_887842 | 41.4 | 1.10E-19 | P39739 | FLIS_BACSU | Flagellar secretion chaperone FliS |
| [denovogenes]_892622 | 40.5 | 1.10E-19 | P39739 | FLIS_BACSU | Flagellar secretion chaperone FliS |
| [denovogenes]_913690 | 40.2 | 2.20E-15 | P69976 | SCTL_YERPE | Type 3 secretion system stator protein |
| [denovogenes]_914104 | 40.5 | 4.10E-14 | Q2YJ78 | VIRB8_BRUA2 | Type IV secretion system protein virB8 |
| [denovogenes]_935262 | 95.7 | 2.30E-54 | Q46833 | YGHE_ECOLI | Type II secretion system L-type protein YghE |
| [denovogenes]_954021 | 42.6 | 2.30E-06 | P31733 | GSPG_AERHY | Type II secretion system core protein G |
| [denovogenes]_966238 | 40 | 6.70E-06 | P45773 | GSPG_VIBCH | Type II secretion system core protein G |
| [denovogenes]_972240 | 47.5 | 1.50E-08 | Q00514 | GSPG_PSEAE | Type II secretion system core protein G |
| [denovogenes]_973695 | 42.5 | 1.60E-18 | P39739 | FLIS_BACSU | Flagellar secretion chaperone FliS |
| [denovogenes]_975494 | 79 | 1.70E-41 | P31586 | GSPG_PECCC | Type II secretion system core protein G |
| [denovogenes]_980914 | 44.5 | 5.70E-21 | P39739 | FLIS_BACSU | Flagellar secretion chaperone FliS |
| [denovogenes]_1263909 | 56.8 | 1.20E-21 | P15752 | GSPM_KLEPN | Type II secretion system protein M |
| [denovogenes]_1264092 | 53.1 | 1.70E-20 | P15747 | GSPH_KLEPN | Type II secretion system protein H |
| [denovogenes]_1300300 | 68.8 | 1.40E-24 | P26609 | FLIS_SALTY | Flagellar secretion chaperone FliS |
| [denovogenes]_1304640 | 97.6 | 1.40E-43 | A0A4C3GMC1 | GSPJ_ECOLX | Type II secretion system protein J |
| [denovogenes]_1342017 | 42.7 | 4.60E-12 | P39739 | FLIS_BACSU | Flagellar secretion chaperone FliS |
| [denovogenes]_1692153 | 96.1 | 2.40E-19 | Q8VPC3 | GSPI_ECOLX | Type II secretion system protein I |
| [denovogenes]_1782445 | 44 | 1.90E-190 | Q9RPY1 | VIRB4_BRUSU | Type IV secretion system protein virB4 |
| [denovogenes]_1793212 | 40.1 | 1.50E-154 | Q9I737 | VGR1B_PSEAE | Type VI secretion system spike protein VgrG1b |
| [denovogenes]_1802541 | 43 | 5.10E-88 | Q00512 | GSPE_PSEAE | Type II secretion system protein E |
| [denovogenes]_1803389 | 42.8 | 2.50E-87 | Q00512 | GSPE_PSEAE | Type II secretion system protein E |
| [denovogenes]_1803559 | 44.2 | 4.10E-138 | E0SIS4 | VGRGB_DICD3 | Type VI secretion system protein VgrGB |
| [denovogenes]_1806655 | 43.1 | 1.60E-123 | E0SAL0 | VGRGA_DICD3 | Type VI secretion system protein VgrGA |
| [denovogenes]_17009 | 55.7 | 1.30E-106 | P15751 | GSPL_KLEPN | Type II secretion system protein L |
| [denovogenes]_22269 | 51.9 | 6.00E-88 | Q8YDY9 | VIRBB_BRUME | Type IV secretion system protein VirB11 |
| [denovogenes]_1816416 | 40.7 | 1.30E-133 | Q0PZF7 | VGRG3_AERHY | Type VI secretion system spike protein VgrG3 |
| [denovogenes]_1822577 | 43.5 | 9.10E-97 | E0SIS4 | VGRGB_DICD3 | Type VI secretion system protein VgrGB |
| [denovogenes]_1822896 | 43.8 | 5.10E-140 | E0SIS4 | VGRGB_DICD3 | Type VI secretion system protein VgrGB |
| [denovogenes]_1825065 | 58.1 | 6.40E-188 | Q03024 | APRD_PSEAE | Alkaline protease secretion ATP-binding protein AprD |
| [denovogenes]_1826144 | 44.8 | 1.70E-87 | P31703 | GSPE_PECCC | Type II secretion system protein E |
| [denovogenes]_1834521 | 58.4 | 3.30E-189 | Q03024 | APRD_PSEAE | Alkaline protease secretion ATP-binding protein AprD |
| [denovogenes]_1836689 | 43.5 | 2.40E-99 | P45759 | GSPE_ECOLI | Type II secretion system protein E |
| [denovogenes]_1838882 | 44.3 | 5.40E-96 | Q01566 | GSPE_DICD3 | Type II secretion system protein E |
| [denovogenes]_1839111 | 45.5 | 1.00E-94 | E0SAL0 | VGRGA_DICD3 | Type VI secretion system protein VgrGA |
| [denovogenes]_1839429 | 41.8 | 1.00E-94 | P37093 | GSPE_VIBCH | Type II secretion system ATPase E |
| [denovogenes]_1839460 | 40.2 | 1.70E-102 | P31741 | GSPE_AERHY | Type II secretion system protein E |
| [denovogenes]_1839495 | 52.9 | 1.10E-160 | P35672 | SCTC1_SALTY | SPI-1 type 3 secretion system secretin |
| [denovogenes]_1841409 | 41.8 | 1.10E-93 | P31741 | GSPE_AERHY | Type II secretion system protein E |
| [denovogenes]_1841596 | 41.5 | 2.50E-93 | P37093 | GSPE_VIBCH | Type II secretion system ATPase E |
| [denovogenes]_1841805 | 41.3 | 3.20E-96 | Q9I5N9 | HXCR_PSEAE | Type II secretion system protein HxcR |
| [denovogenes]_1841865 | 41.7 | 2.90E-97 | Q9I5N9 | HXCR_PSEAE | Type II secretion system protein HxcR |
| [denovogenes]_1841924 | 41.5 | 2.40E-96 | Q9I5N9 | HXCR_PSEAE | Type II secretion system protein HxcR |
| [denovogenes]_1842415 | 44.1 | 3.10E-96 | P45759 | GSPE_ECOLI | Putative type II secretion system protein E |
| [denovogenes]_1844121 | 42.5 | 1.80E-51 | P9WPI3 | ECCA3_MYCTU | ESX-3 secretion system protein EccA3 |
| [denovogenes]_1844442 | 40.5 | 1.10E-51 | Q9CD28 | ECCA3_MYCLE | ESX-3 secretion system protein EccA3 |
| [denovogenes]_1844618 | 42.8 | 4.20E-93 | P15645 | GSPE_KLEPN | Type II secretion system protein E |
| [denovogenes]_1846083 | 40.3 | 1.00E-91 | P45759 | GSPE_ECOLI | Putative type II secretion system protein E |
| [denovogenes]_1847066 | 40.1 | 1.20E-92 | P31741 | GSPE_AERHY | Type II secretion system protein E |
| [denovogenes]_1847258 | 44 | 6.20E-89 | Q9I5N9 | HXCR_PSEAE | Type II secretion system protein HxcR |
| [denovogenes]_1847546 | 40.4 | 2.50E-66 | Q00512 | GSPE_PSEAE | Type II secretion system protein E |
| [denovogenes]_1863176 | 43.3 | 1.90E-95 | P31741 | GSPE_AERHY | Type II secretion system protein E |
| [denovogenes]_1864511 | 40.4 | 1.10E-98 | Q9I748 | TSSC1_PSEAE | Type VI secretion system sheath protein TssC1 |
| [denovogenes]_1864573 | 42.3 | 2.60E-97 | Q9I748 | TSSC1_PSEAE | Type VI secretion system sheath protein TssC1 |
| [denovogenes]_1864624 | 40.1 | 6.90E-98 | Q9I748 | TSSC1_PSEAE | Type VI secretion system sheath protein TssC1 |
| [denovogenes]_1873730 | 74.2 | 3.20E-217 | Q9I748 | TSSC1_PSEAE | Type VI secretion system sheath protein TssC1 |
| [denovogenes]_1873733 | 72.7 | 8.40E-218 | Q9I748 | TSSC1_PSEAE | Type VI secretion system sheath protein TssC1 |
| [denovogenes]_1874742 | 86.3 | 1.20E-240 | P15645 | GSPE_KLEPN | Type II secretion system protein E |
| [denovogenes]_1875089 | 43.4 | 5.20E-111 | P0C525 | VIRB4_BRUAB | Type IV secretion system protein virB4 |
| [denovogenes]_1875142 | 67.6 | 3.00E-183 | P37093 | GSPE_VIBCH | Type II secretion system ATPase E |
| [denovogenes]_1877270 | 42.5 | 3.40E-102 | Q9I748 | TSSC1_PSEAE | Type VI secretion system sheath protein TssC1 |
| [denovogenes]_1877740 | 99.8 | 1.50E-275 | P45759 | GSPE_ECOLI | Putative type II secretion system protein E |
| [denovogenes]_1878244 | 46.3 | 2.50E-121 | Q9I748 | TSSC1_PSEAE | Type VI secretion system sheath protein TssC1 |
| [denovogenes]_1879390 | 40.3 | 4.30E-97 | Q9I748 | TSSC1_PSEAE | Type VI secretion system sheath protein TssC1 |
| [denovogenes]_1880327 | 100 | 2.40E-286 | P45756 | GSPA_ECOLI | Putative general secretion pathway protein A |
| [denovogenes]_1886091 | 45.5 | 2.40E-92 | Q01566 | GSPE_DICD3 | Type II secretion system protein E |
| [denovogenes]_1889892 | 45.8 | 3.10E-92 | P45759 | GSPE_ECOLI | Putative type II secretion system protein E |
| [denovogenes]_1891118 | 47.7 | 4.70E-93 | P37093 | GSPE_VIBCH | Type II secretion system ATPase E |
| [denovogenes]_1906448 | 49.4 | 3.30E-91 | Q9I5N9 | HXCR_PSEAE | Type II secretion system protein HxcR |
| [denovogenes]_1907778 | 43.9 | 7.50E-104 | Q9I744 | TSSF1_PSEAE | Type VI secretion system component TssF1 |
| [denovogenes]_1909292 | 42.5 | 1.10E-86 | P31703 | GSPE_PECCC | Type II secretion system protein E |
| [denovogenes]_1915313 | 46.7 | 3.20E-107 | Q00512 | GSPE_PSEAE | Type II secretion system protein E |
| [denovogenes]_1916064 | 42.5 | 1.70E-89 | P31703 | GSPE_PECCC | Type II secretion system protein E |
| [denovogenes]_1916892 | 41.5 | 1.80E-94 | Q9I753 | TSSK1_PSEAE | Type VI secretion system baseplate component TssK1 |
| [denovogenes]_1922376 | 45.2 | 2.00E-98 | P31741 | GSPE_AERHY | Type II secretion system protein E |
| [denovogenes]_1923260 | 43.8 | 2.50E-96 | P37093 | GSPE_VIBCH | Type II secretion system ATPase E |
| [denovogenes]_1925345 | 64.5 | 6.60E-150 | P0A1B9 | SCTN1_SALTY | SPI-1 type 3 secretion system ATPase |
| [denovogenes]_1925999 | 69.6 | 2.70E-159 | P40290 | SCTN_YEREN | Type 3 secretion system ATPase |
| [denovogenes]_1926641 | 68.4 | 9.50E-165 | P40290 | SCTN_YEREN | Type 3 secretion system ATPase |
| [denovogenes]_1928391 | 48.5 | 2.40E-112 | E0SIS4 | VGRGB_DICD3 | Putative type VI secretion system protein VgrGB |
| [denovogenes]_1928513 | 46.6 | 4.20E-96 | Q01566 | GSPE_DICD3 | Type II secretion system protein E |
| [denovogenes]_1929781 | 45.6 | 4.60E-87 | P31742 | GSPE_XANCP | Type II secretion system protein E |
| [denovogenes]_1930663 | 43.8 | 2.10E-87 | Q00512 | GSPE_PSEAE | Type II secretion system protein E |
| [denovogenes]_1931276 | 43.9 | 5.70E-69 | Q2YJ82 | VIRBB_BRUA2 | Type IV secretion system protein VirB11 |
| [denovogenes]_1943477 | 41.5 | 2.20E-89 | P31702 | GSPE_DICCH | Type II secretion system protein E |
| [denovogenes]_1944104 | 45.7 | 1.80E-91 | P45759 | GSPE_ECOLI | Putative type II secretion system protein E |
| [denovogenes]_1944476 | 44.2 | 4.50E-79 | Q9I748 | TSSC1_PSEAE | Type VI secretion system sheath protein TssC1 |
| [denovogenes]_1946079 | 46.1 | 1.50E-90 | Q01566 | GSPE_DICD3 | Type II secretion system protein E |
| [denovogenes]_1946902 | 46.2 | 2.60E-95 | E0SIS4 | VGRGB_DICD3 | Type VI secretion system protein VgrGB |
| [denovogenes]_1947452 | 41.8 | 5.20E-27 | Q7VSX3 | PTLH_BORPE | Type IV secretion system protein PtlH |
| [denovogenes]_1950770 | 43.4 | 7.10E-61 | Q0GK35 | VIRBA_BRUO2 | Type IV secretion system protein virB10 |
| [denovogenes]_1955982 | 46.3 | 2.60E-92 | Q00512 | GSPE_PSEAE | Type II secretion system protein E |
| [denovogenes]_1957231 | 40 | 2.10E-41 | Q9RPX5 | VIRBA_BRUSU | Type IV secretion system protein virB10 |
| [denovogenes]_1957809 | 45.4 | 2.30E-88 | Q9I5N9 | HXCR_PSEAE | Type II secretion system protein HxcR |
| [denovogenes]_1960440 | 56.5 | 1.40E-122 | P45780 | GSPF_VIBCH | Type II secretion system protein F |
| [denovogenes]_1962255 | 44.4 | 4.60E-81 | Q00512 | GSPE_PSEAE | Type II secretion system protein E |
| [denovogenes]_1962324 | 41.8 | 5.90E-28 | Q7W2T7 | PTLH_BORPA | Type IV secretion system protein PtlH homolog |
| [denovogenes]_1963319 | 78.7 | 5.00E-168 | P15745 | GSPF_KLEPN | Type II secretion system protein F |
| [denovogenes]_1970957 | 100 | 3.00E-218 | P41441 | GSPF_ECOLI | Putative type II secretion system protein F |
| [denovogenes]_1972773 | 54.4 | 1.00E-112 | P15751 | GSPL_KLEPN | Type II secretion system protein L |
| [denovogenes]_1979013 | 44.1 | 1.20E-81 | Q00512 | GSPE_PSEAE | Type II secretion system protein E |
| [denovogenes]_1979553 | 48.7 | 1.70E-93 | Q00512 | GSPE_PSEAE | Type II secretion system protein E |
| [denovogenes]_1979570 | 99.3 | 3.10E-159 | Q46833 | YGHE_ECOLI | Type II secretion system L-type protein YghE |
| [denovogenes]_1980086 | 47.9 | 2.80E-91 | Q00512 | GSPE_PSEAE | Type II secretion system protein E |
| [denovogenes]_1980268 | 48.9 | 6.00E-94 | Q00512 | GSPE_PSEAE | Type II secretion system protein E |
| [denovogenes]_1980518 | 44.8 | 1.40E-82 | Q00512 | GSPE_PSEAE | Type II secretion system protein E |
| [denovogenes]_1982779 | 90 | 1.00E-194 | P15645 | GSPE_KLEPN | Type II secretion system protein E |
| [denovogenes]_1983873 | 40.1 | 5.60E-60 | Q01566 | GSPE_DICD3 | Type II secretion system protein E |
| [denovogenes]_31980 | 52.6 | 2.80E-90 | Q8YDY9 | VIRBB_BRUME | Type IV secretion system protein VirB11 |
| [denovogenes]_32176 | 52.4 | 4.10E-89 | Q8YDY9 | VIRBB_BRUME | Type IV secretion system protein VirB11 |
| [denovogenes]_32498 | 52.6 | 4.80E-90 | Q2YJ82 | VIRBB_BRUA2 | Type IV secretion system protein VirB11 |
| [denovogenes]_39564 | 47.1 | 2.10E-90 | Q00512 | GSPE_PSEAE | Type II secretion system protein E |
| [denovogenes]_40660 | 47.5 | 1.40E-86 | P37093 | GSPE_VIBCH | Type II secretion system ATPase E |
| [denovogenes]_43049 | 43.3 | 7.30E-83 | P37093 | GSPE_VIBCH | Type II secretion system ATPase E |
| [denovogenes]_45262 | 48.3 | 1.10E-91 | Q9I748 | TSSC1_PSEAE | Type VI secretion system sheath protein TssC1 |
| [denovogenes]_45886 | 42.2 | 2.00E-72 | Q00512 | GSPE_PSEAE | Type II secretion system protein E |
| [denovogenes]_48464 | 46.5 | 2.00E-72 | Q9I5N9 | HXCR_PSEAE | Type II secretion system protein HxcR |
| [denovogenes]_48513 | 50.1 | 4.40E-88 | Q8YDY9 | VIRBB_BRUME | Type IV secretion system protein VirB11 |
| [denovogenes]_51049 | 46.9 | 5.00E-68 | E0SAL0 | VGRGA_DICD3 | Type VI secretion system protein VgrGA |
| [denovogenes]_52206 | 56.8 | 7.60E-101 | Q8YDY9 | VIRBB_BRUME | Type IV secretion system protein VirB11 |
| [denovogenes]_1984991 | 54 | 5.90E-110 | P15751 | GSPL_KLEPN | Type II secretion system protein L |
| [denovogenes]_1985225 | 47.6 | 7.50E-105 | Q00512 | GSPE_PSEAE | Type II secretion system protein E |
| [denovogenes]_1985795 | 99.7 | 4.10E-228 | P45763 | GSPL_ECOLI | Putative type II secretion system protein L |
| [denovogenes]_59438 | 41.8 | 1.40E-62 | Q8FXK7 | VIRBB_BRUSU | Type IV secretion system protein VirB11 |
| [denovogenes]_62542 | 40 | 9.30E-59 | Q8FXK7 | VIRBB_BRUSU | Type IV secretion system protein VirB11 |
| [denovogenes]_67737 | 49.8 | 7.20E-72 | P37093 | GSPE_VIBCH | Type II secretion system ATPase E |
| [denovogenes]_67836 | 49.4 | 3.60E-71 | P37093 | GSPE_VIBCH | Type II secretion system ATPase E |
| [denovogenes]_67973 | 48.9 | 7.20E-72 | Q01566 | GSPE_DICD3 | Type II secretion system protein E |
| [denovogenes]_71635 | 46.4 | 1.10E-75 | Q9KNE7 | VGRG2_VIBCH | Type VI secretion system spike protein VgrG2 |
| [denovogenes]_78912 | 41.1 | 7.40E-45 | A0QNI9 | ECCA1_MYCS2 | ESX-1 secretion system protein EccA1 |
| [denovogenes]_82742 | 100 | 5.00E-187 | P45762 | GSPK_ECOLI | Putative type II secretion system protein K |
| [denovogenes]_85600 | 72.7 | 1.60E-132 | P15750 | GSPK_KLEPN | Type II secretion system protein K |
| [denovogenes]_87050 | 42.9 | 6.60E-62 | P45781 | EPSK_VIBCH | Type II secretion system protein K |
| [denovogenes]_97712 | 94.7 | 1.10E-167 | Q46834 | YGHF_ECOLI | Type II secretion system C-type protein YghF |
| [denovogenes]_98225 | 92.5 | 3.20E-162 | E3PJ87 | GSPC2_ECOH1 | Type II secretion system protein C 2 |
| [denovogenes]_100332 | 71.7 | 2.40E-125 | P15750 | GSPK_KLEPN | Type II secretion system protein K |
| [denovogenes]_101911 | 60.6 | 5.40E-101 | P31743 | GSPF_AERHY | Type II secretion system protein F |
| [denovogenes]_109757 | 55.9 | 6.30E-94 | P41441 | GSPF_ECOLI | Putative type II secretion system protein F |
| [denovogenes]_118713 | 46.7 | 2.80E-70 | Q00512 | GSPE_PSEAE | Type II secretion system protein E |
| [denovogenes]_121486 | 45.3 | 4.80E-54 | Q9I5N9 | HXCR_PSEAE | Type II secretion system protein HxcR |
| [denovogenes]_123239 | 40.3 | 8.70E-56 | Q9I753 | TSSK1_PSEAE | Type VI secretion system baseplate component TssK1 |
| [denovogenes]_124533 | 61.6 | 2.60E-105 | P40290 | SCTN_YEREN | Type 3 secretion system ATPase |
| [denovogenes]_138789 | 74.6 | 1.60E-123 | P45759 | GSPE_ECOLI | Type II secretion system protein E |
| [denovogenes]_142548 | 47.9 | 2.20E-72 | P31703 | GSPE_PECCC | Type II secretion system protein E |
| [denovogenes]_157949 | 55.8 | 7.60E-78 | Q2YJ82 | VIRBB_BRUA2 | Type IV secretion system protein VirB11 |
| [denovogenes]_157994 | 44.2 | 1.40E-50 | Q0GK35 | VIRBA_BRUO2 | Type IV secretion system protein virB10 |


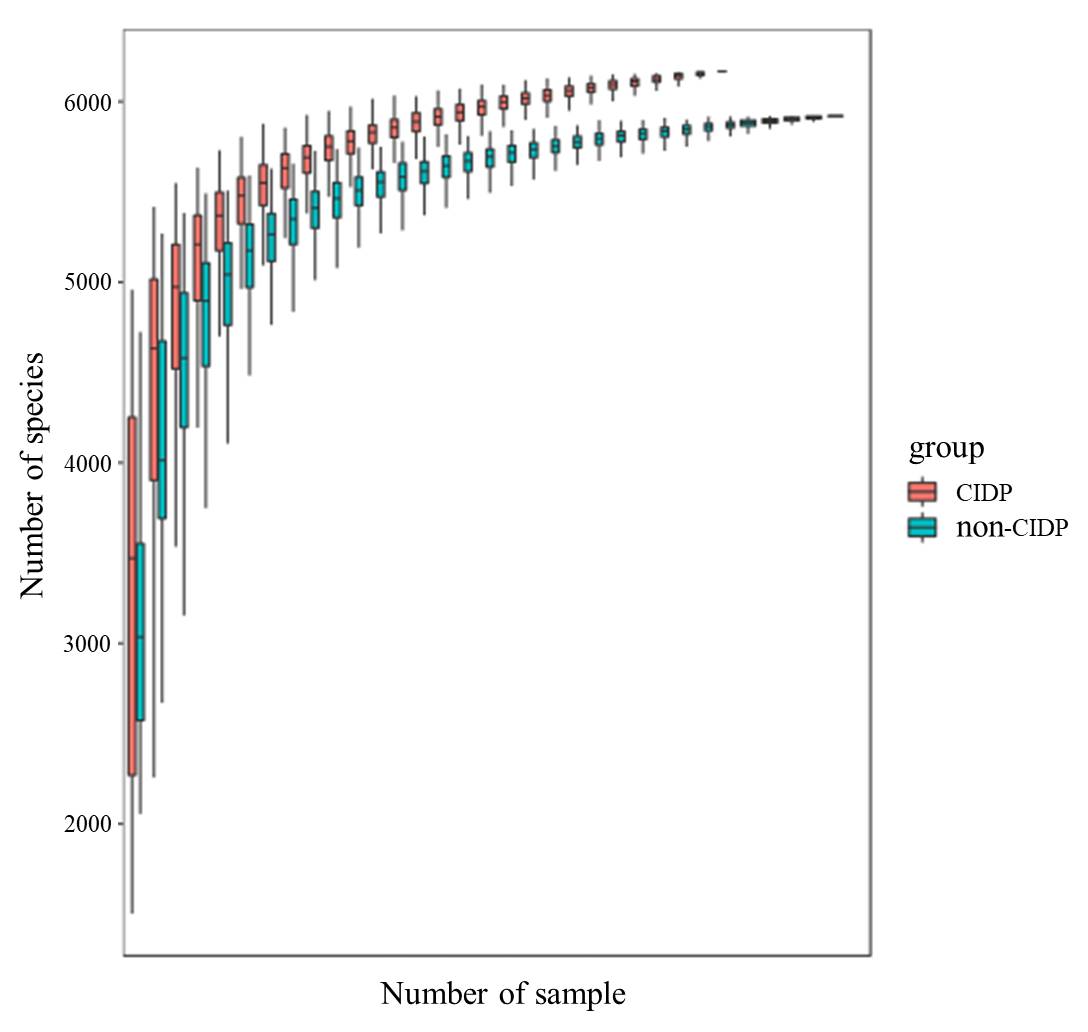


Figure S1 Rarefaction curve Boxplot. Abscissa represents for sample size while ordinate represents for number of species in sample. Diversity is limited when sample size is small, which is not reliable to represent for the entire microbiota structure. When rarefaction curve tends to be steady, it indicates that the sampling quantity is sufficient.


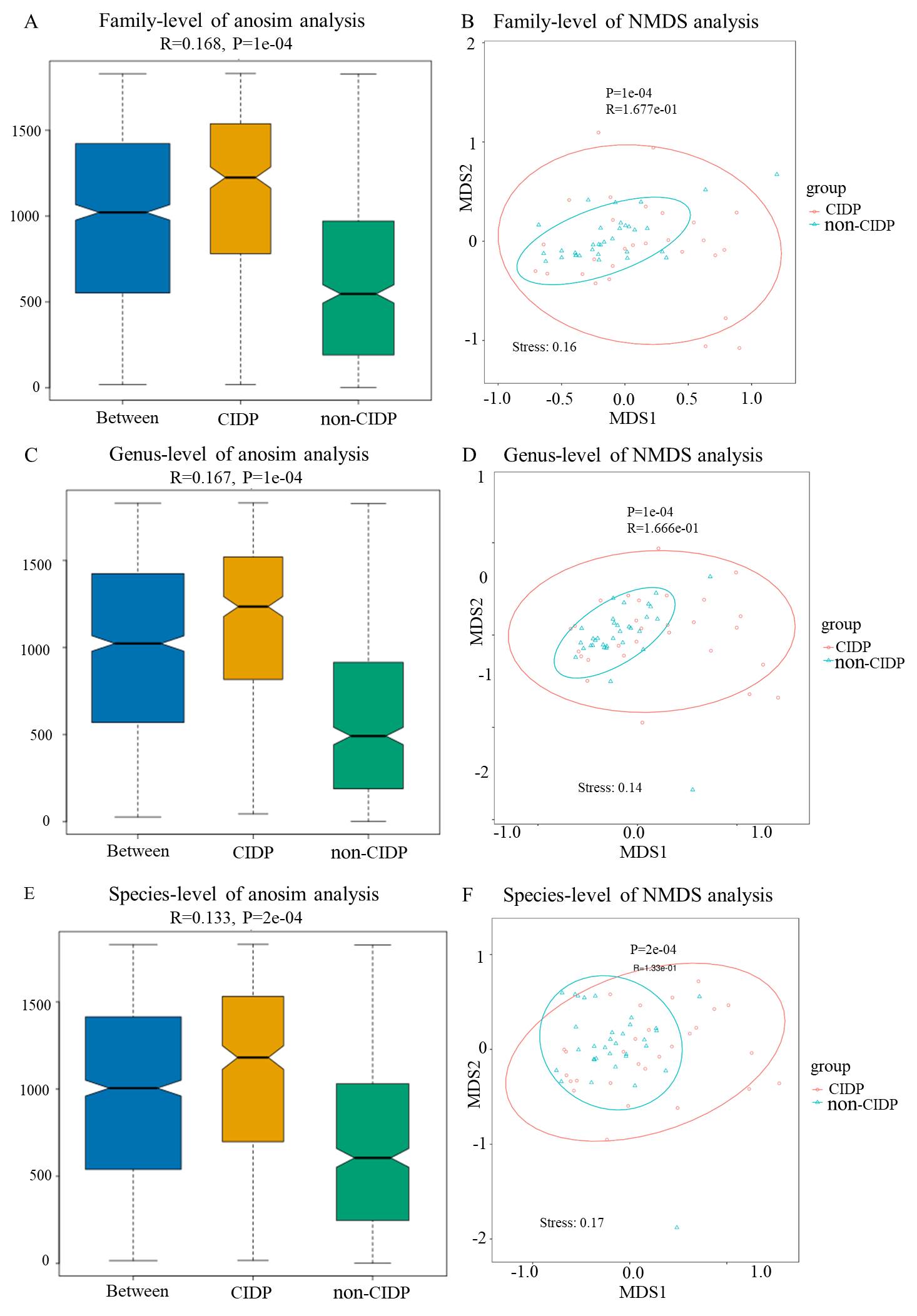


Figure S2 NMDS and Anosim analysis. Anosim analysis at the family-level (A), genus-level (C) and the species-level (E). Between represents the distance between CIDP and non-CIDP groups, and the remaining boxes represent the distance within the corresponding group. NMDS analysis at the family-level (B), genus-level (D) and the species-level (F). Scales on X-axis and Y-axis on NMDS graph are the projection axes of samples in 2D.


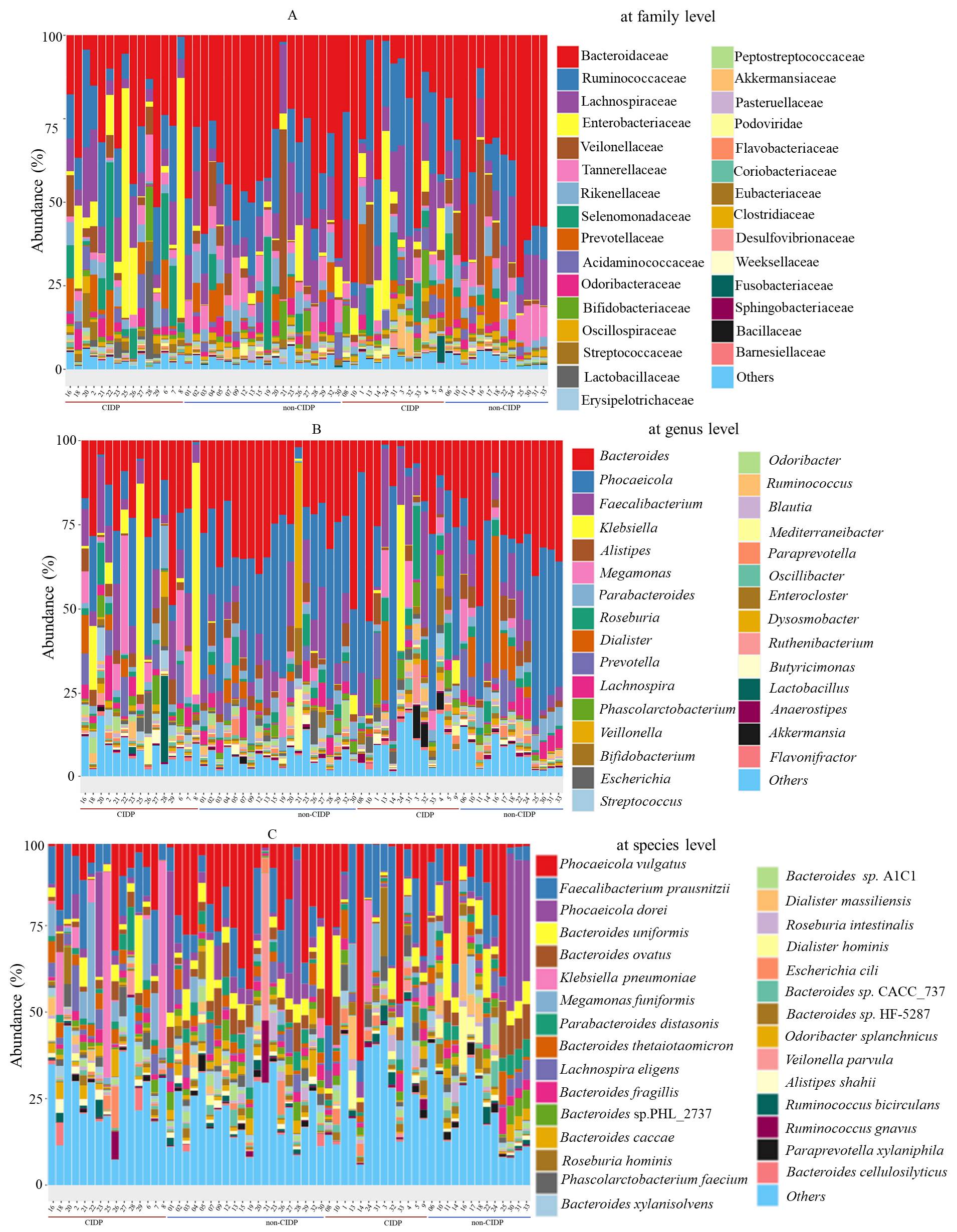


Figure S3 Microbial classifications and abundance in individual samples shown at family-level (A), genus-level (B) and the species-level (C).


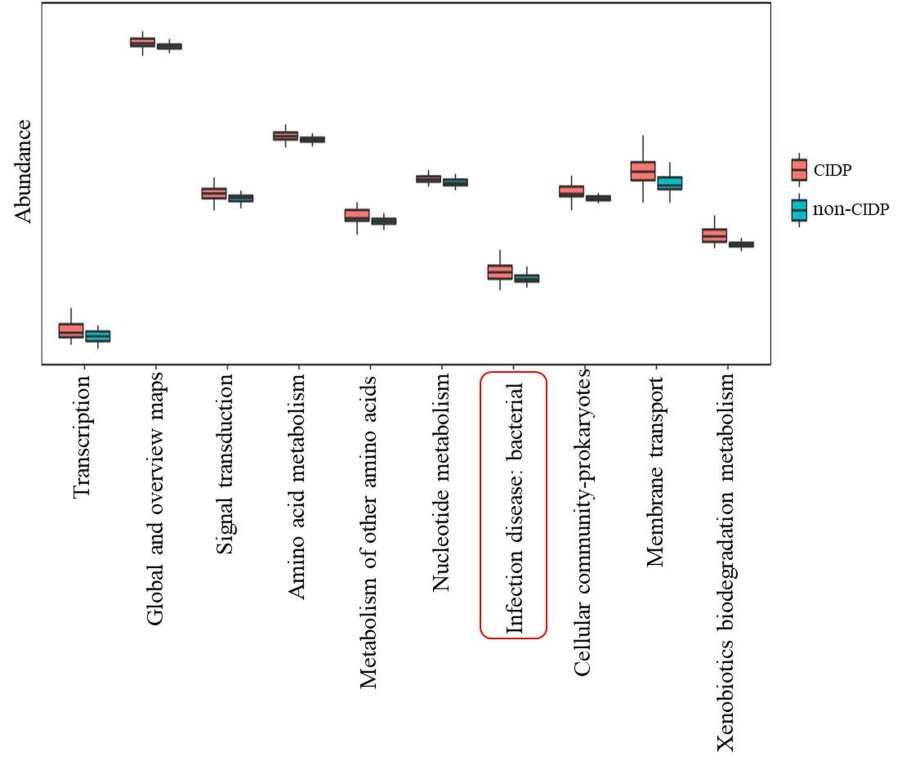


Figure S4 Box plot of enriched pathways in CIDP of KEGG secondary classification. The gene number can be viewed on the X-axis, the secondary classification can be viewed on Y-axis.


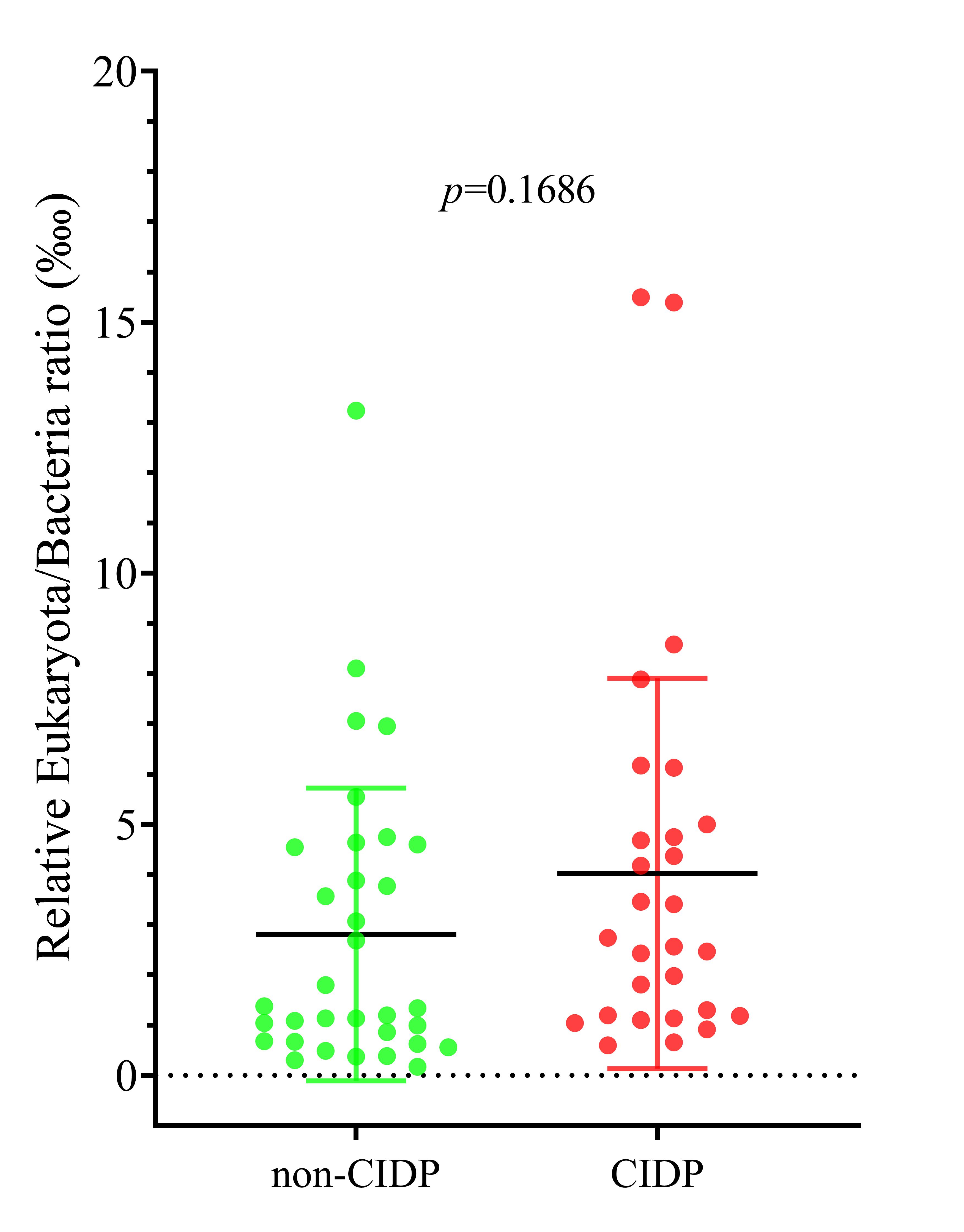


Figure S5 Comparisons of the relative Eukaryota-Bacteria ratio in the stool of non-CIDP and CIDP group. Data are shown as mean ± SEM.
